# Human *KIT*^+^ myeloid cells facilitate visceral metastasis by melanoma

**DOI:** 10.1084/jem.20182163

**Published:** 2021-04-15

**Authors:** Chun I. Yu, Jan Martinek, Te-Chia Wu, Kyung In Kim, Joshy George, Elaheh Ahmadzadeh, Rick Maser, Florentina Marches, Patrick Metang, Pierre Authie, Vanessa K.P. Oliveira, Victor G. Wang, Jeffrey H. Chuang, Paul Robson, Jacques Banchereau, Karolina Palucka

**Affiliations:** 1 The Jackson Laboratory for Genomic Medicine, Farmington, CT; 2 The Jackson Laboratory for Mammalian Genetics, Bar Harbor, ME; 3 Department of Genetics and Genome Sciences, University of Connecticut Health Center, Farmington, CT

## Abstract

Metastasis of melanoma significantly worsens prognosis; thus, therapeutic interventions that prevent metastasis could improve patient outcomes. Here, we show using humanized mice that colonization of distant visceral organs with melanoma is dependent upon a human CD33^+^CD11b^+^CD117^+^ progenitor cell subset comprising <4% of the human CD45^+^ leukocytes. Metastatic tumor-infiltrating CD33^+^ cells from patients and humanized (h)NSG-SGM3 mice showed converging transcriptional profiles. Single-cell RNA-seq analysis identified a gene signature of a *KIT*/CD117–expressing CD33^+^ subset that correlated with decreased overall survival in a TCGA melanoma cohort. Thus, human CD33^+^CD11b^+^CD117^+^ myeloid cells represent a novel candidate biomarker as well as a therapeutic target for metastatic melanoma.

## Teaser

Colonization of visceral organs with melanoma in humanized NSG-SGM3 mice is dependent upon human CD33^+^CD11b^+^CD117^+^ progenitor cells. A gene signature of *KIT*/CD117–expressing CD33^+^ subset correlates with decreased overall survival in TCGA melanoma samples and represents a novel candidate biomarker.

## Graphical Abstract

**Figure:**
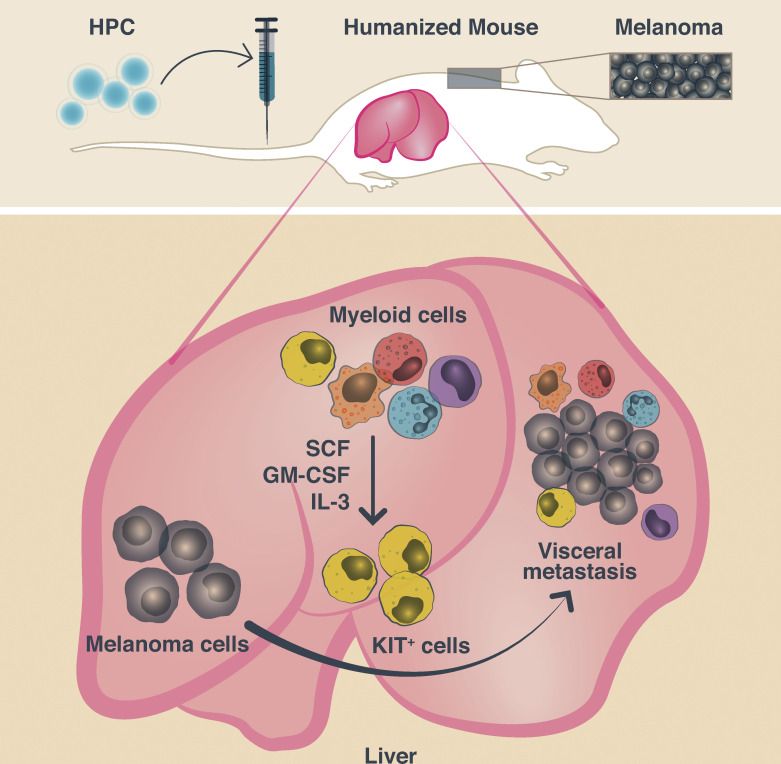

## Introduction

Mechanisms sustaining the growth of human melanoma in distant organs remain poorly defined. This is important because metastatic dissemination remains a major clinical challenge (Eggermont et al., 2016). Melanoma can metastasize to all organs (Gupta and Brasfield, 1964; Einhorn et al., 1974; Meyer and Stolbach, 1978; Nathanson et al., 1967; Patel et al., 1978), and patients with nonpulmonary visceral metastasis, including liver, have the worst prognosis (Balch et al., 2009). Improved survival has been documented for patients with metastatic melanoma treated with B-raf proto-oncogene serine/threonine kinase– and/or mitogen-activated protein kinase kinase–targeted therapies (Flaherty et al., 2016; Long et al., 2017) or with blocking antibodies targeting CTL-associated protein (CTLA)-4 (Hodi et al., 2010) and/or programmed death (PD)-1 (Wolchok et al., 2017). However, a significant fraction of patients do not achieve prolonged survival even in combination therapy trials and succumb to treatment-resistant metastatic disease (Wolchok et al., 2017).

Dissemination and growth in distant organs are driven by a complex interplay between cancer cells and their microenvironment. A number of cell types of myeloid lineage have been implicated in cancer metastasis (Garner and de Visser, 2020). In the mouse, neutrophils are engaged in various steps of metastasis (Jaillon et al., 2020), including support of cancer cell proliferation and survival (Acharyya et al., 2012; Ouzounova et al., 2017; Szczerba et al., 2019), angiogenesis (Bekes et al., 2011; Nozawa et al., 2006), increased extravasation of disseminated cancer cells (Spiegel et al., 2016), and inhibition of natural killer (NK) cell– and CTL-mediated cancer cell clearance (Coffelt et al., 2015; Spiegel et al., 2016). Immature myeloid cells that have not completed differentiation and are able to exert suppressive effects on adaptive immunity (i.e., myeloid-derived suppressor cells) promote invasion, angiogenesis, and metastasis formation (Datta et al., 2019). Furthermore, tumor-associated macrophages promote development of metastatic disease by means of supporting tumor growth in target tissues and escape from NK and T cells (Etzerodt et al., 2019; Lewis et al., 2016; Mantovani et al., 2017; Ruffell and Coussens, 2015). Thus, myeloid cells could serve as putative therapeutic targets. Their remarkable heterogeneity makes an in-depth characterization a major goal, where in vivo studies would be most informative. However, differences in myeloid cells and their downstream signaling pathways between humans and mice (Hagai et al., 2018; Kanazawa, 2007; Mestas and Hughes, 2004; Williams et al., 2010) make it uncertain to directly extrapolate mouse in vivo data into humans. Here, using a transplantable model of human melanoma in humanized mice, we find that CD117^+^CD11b^+^CD33^+^ myeloid cells support melanoma growth in distant organs.

## Results and discussion

### Metastatic melanoma tumors in patients and in humanized mice are infiltrated with CD33^+^ myeloid cells

**Figure 1.**
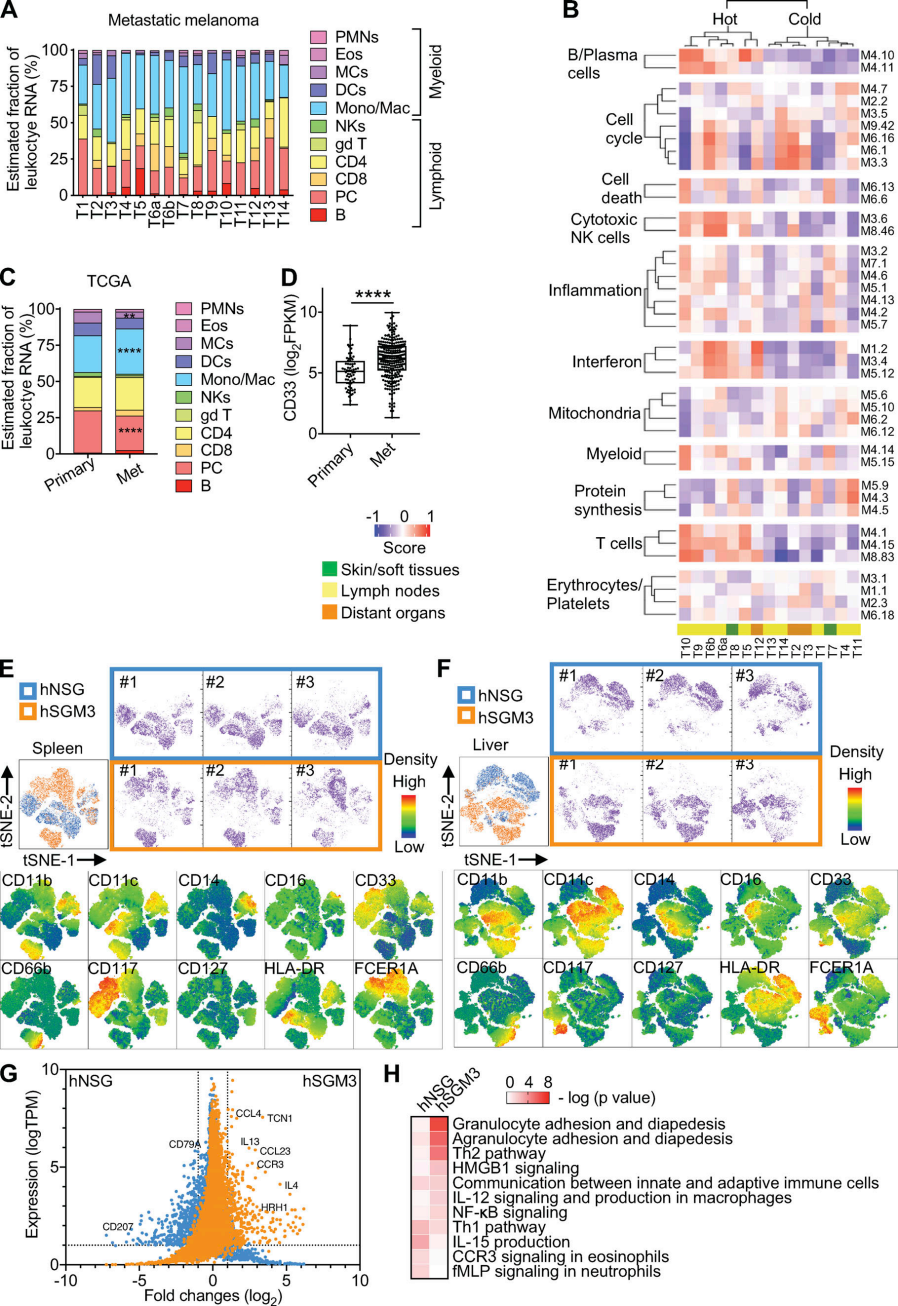
**Human myeloid cells in metastatic melanoma tumors and hNSG-SGM3 mice. (A)** CIBERSORT analysis of RNA-seq data from 14 metastatic patient melanoma tumors (two samples from different areas of one patient tumor). **(B)** GSVA score of patient tumors using defined modules as gene sets. **(C)** CIBERSORT analysis of RNA-seq data from TCGA primary (*n* = 66) and metastatic (Met) melanoma (*n* = 264) tumors. Values are mean percentage. Two-way ANOVA with Bonferroni’s multiple comparisons test. ****, P < 0.0001; **, P < 0.01. **(D)** CD33 expression in TCGA primary and metastatic melanoma tumors. P**= 2.3 × 10^−7^ Wilcoxon test. The error bar is the SD. **(E)** hCD33^+^ cells from the spleen of three hNSG (blue) and three hNSG-SGM3 (orange) mice were gated and subjected to t-distributed stochastic neighbor embedding (tSNE) reduction. Indicated markers were color-mapped from blue (low density) to red (high density) into the tSNE map. **(F)** tSNE plots of hCD33^+^ cells from the liver as analyzed in E.** (G)** Bulk RNA-seq of hCD33^+^ cells enriched and pooled from the spleen and liver of hNSG and hNSG-SGM3 mice. DEGs were illustrated in the dot plot using log_2_ fold change as the x axis and TPM of hNSG (blue) or hNSG-SGM3 (orange) as the y axis with criteria of logTPM expression ≥1 and absolute log2 fold change ≥1 (dotted lines). **(H)** Heatmap comparing IPA on the DEGs in CD33^+^ cells from hNSG and hNSG-SGM3 mice for immune canonical pathway analysis. Eos, eosinophils; DCs, dendritic cells; MCs, mast cells; gd T, γΔ T cells; Mono/Mac, monocyte/macrophage; PC, plasma cells; PMN, polymorphonuclear leukocytes. fMLPN, formyl-L-methionyl-L-leucyl-phenylalanine.

**Figure S1.**
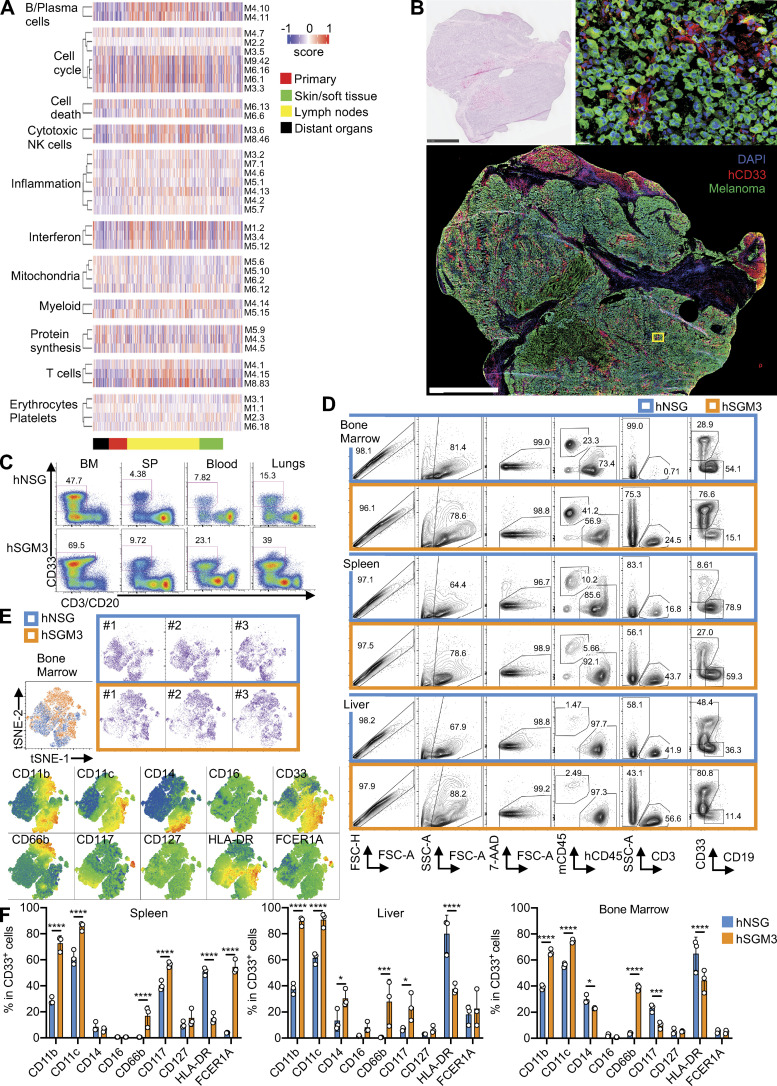
**Human myeloid cells in metastatic melanoma tumors and hNSG-SGM3 mice.** **(A)** Heatmap representing the GSVA enrichment score of primary or metastatic patient melanoma tumors (skin/soft tissues, lymphoid nodes, or distant organs) using defined modules as gene sets. **(B)** H&E and immunofluorescence staining of MART-1/gp100 (green), CD33 (red), and DAPI (blue) on metastatic human melanoma tumor from the liver. Scale bar = 2.5 mm and 20 µm for the zoom-in. **(C)** FACS plots illustrate the percentage of hCD33^+^ cells in hNSG and hNSG-SGM3 (labeled hSGM3) mice at 12 wk after engraftment. BM, bone marrow; SP, spleen. **(D)** Single-cell suspension from the bone marrow, spleen, and liver of hNSG or hNSG-SGM3 mice at 12–14 wk after engraftment were analyzed by FACS. FACS plots illustrated the gating strategies for hCD33^+^ cells. **(E)** hCD33^+^ cells from the bone marrow of three hNSG and three hSGM3 mice were gated and subjected to tSNE reduction. Indicated marker was color mapped from blue (low density) to red (high density) into the tSNE map. **(F)** Bar plot of myeloid markers in hCD33^+^ cells from the spleen, liver, and bone marrow of hNSG and hNSG-SGM3 mice. *n* = 3 mice with two-way ANOVA test. The error bar is the SD. ****, P < 0.0001; ***, P < 0.001; and *, P < 0.05. 7-AAD, 7-amino-actinomycin D; FSC-A, forward scatter-A; FSC-H, forward scatter-H; mCD45, mouse CD45; SSC-A, side scatter-A.

To define the landscape of leukocytes in metastatic melanoma, we analyzed transcriptional profiles of metastatic melanoma tumors from 14 patients with RNA sequencing (RNA-seq; Table S1). CIBERSORT, which estimates the fraction of various leukocyte RNA (Newman et al., 2015), revealed that nearly half of the leukocyte transcripts originated from myeloid cells (Fig. 1 A). Modular analysis (Banchereau et al., 2016) revealed several lymphoid modules including B cells (36 genes), T cells (107 genes), and cytotoxic/NK cells (59 genes) as well as IFN modules (138 genes), which together clustered patient samples into two groups (hot and cold tumors; Fig. 1 B and Table S2). Inflammation (876 genes) and myeloid (78 genes) modules were spread across all samples (Fig. 1 B). These findings were confirmed using The Cancer Genome Atlas (TCGA) melanoma cohort (Cancer Genome Atlas Network, 2015) from primary (*n* = 66) and metastatic (*n* = 264) melanoma tumors from different patients (Fig. S1 A). There, CIBERSORT revealed significantly higher monocyte/macrophage–related transcripts in metastatic versus primary tumors (Fig. 1 C). Metastatic sites also showed a significantly higher expression of pan-myeloid marker CD33 (sialic acid binding Ig-like lectin 3; P = 2.3e-07; Fig. 1 D). Immunofluorescence staining revealed the presence of CD33^+^ myeloid cells in close proximity to melanoma cells in patient tumors (Fig. S1 B).

To study the in vivo impact of human CD33^+^ (hCD33^+^) myeloid cells on human melanoma, we turned to nonobese diabetic/severe combined immunodeficient-γ (NSG) mice with transgenic expression of the human hematopoietic cytokines stem cell factor (SCF), GM-CSF, and IL-3 (NSG–SCF GM-CSF IL-3 [SGM3] mice) transplanted with hCD34^+^ hematopoietic progenitor cells (HPCs), which support the development of hCD33^+^ myeloid cells (Fig. S1 C, Table S3, and Table S4; Billerbeck et al., 2011). FACS analysis of hCD33^+^ cells from bone marrow, liver, and spleen at 12 wk after hCD34^+^ HPC transplant using CD11b, CD11c, CD14, CD16, CD66b, CD117, CD127, HLA-DR, and FCER1A markers (Fig. 1, E and F; and Fig. S1, D and E) revealed the qualitative and quantitative differences between humanized (h)NSG and hNSG-SGM3 mice, the latter ones displaying monocytes, granulocytes, and mast cells (Fig. S1 F). The transcriptional profiles of bulk hCD33^+^ cells from the spleen and liver of hNSG and hNSG-SGM3 mice at 12 wk after hCD34^+^ HPC transplant confirmed the phenotypic divergence between the two strains (Fig. 1 G and Table S5), with granulocyte adhesion and diapedesis and Th2 cell pathways significantly enriched in hCD33^+^ cells from hNSG-SGM3 mice (Fig. 1 H).

### hNSG-SGM3 mice support distant organ colonization with melanoma

**Figure 2.**
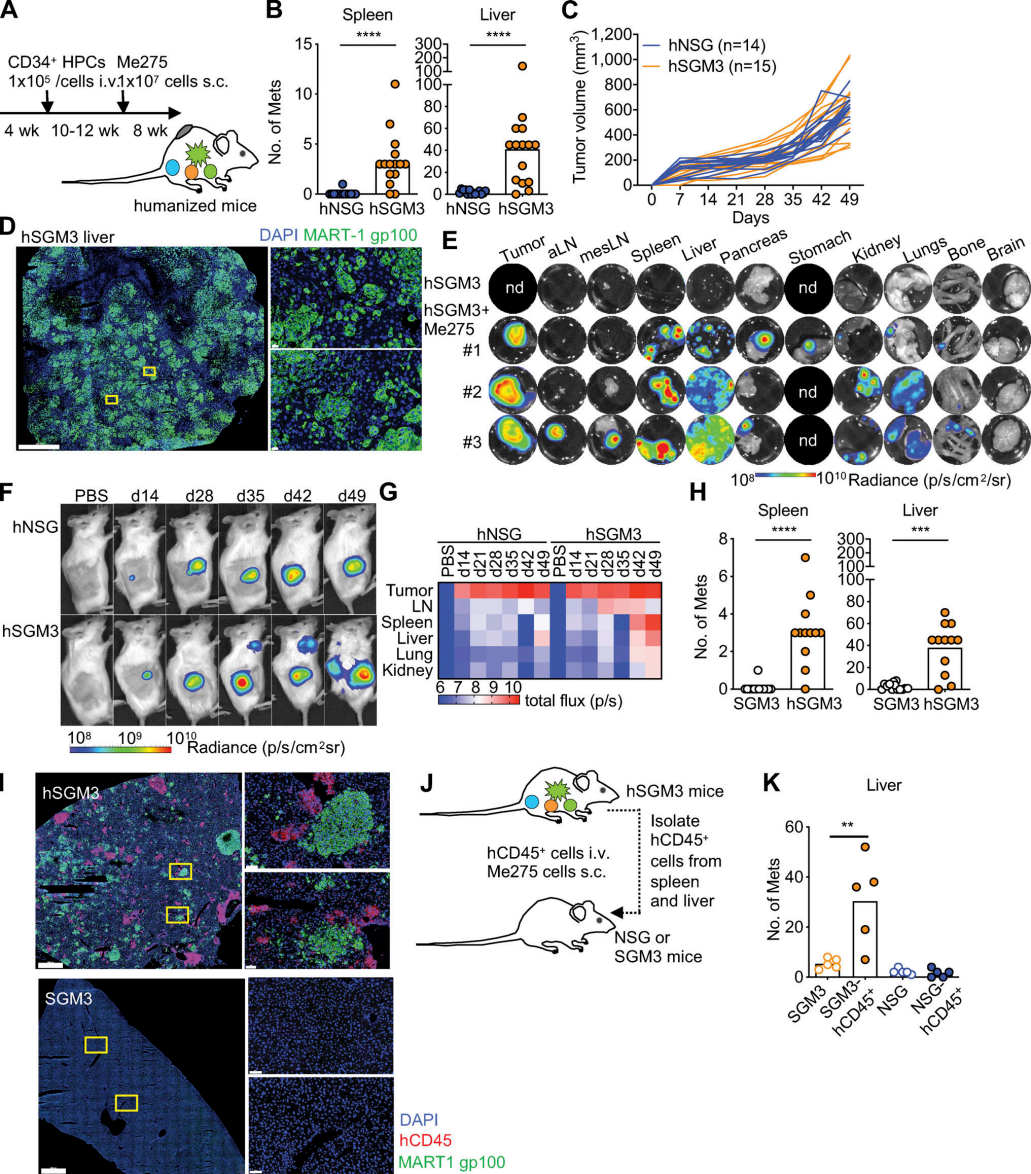
**hNSG-SGM3 mice promote melanoma growth in distant organs. (A)** Mice were engrafted with hCD34^+^ HPCs at 4 wk, implanted with 10^7^ Me275 cells s.c. at 14–16 wk, and analyzed at 22–24 wk of age (7–8 wk after tumor implantation) before succumbing to the disease. **(B)** Number of macroscopic tumors in the spleen and liver of hNSG and hNSG-SGM3 mice. Two-tailed Mann-Whitney test. Data are combined from a total of 14 hNSG and 15 hNSG-SGM3 mice from three independent experiments (i.e., separate mouse cohorts) using two CD34^+^ HPC donors. Mets, metastases. **(C)** Me275 tumor growth in hNSG and hNSG-SGM3 mice as in B. **(D)** Localization of MART-1/gp100 (green) and DAPI (blue) in the liver of hNSG-SGM3 mice at 8 wk. Scale bar = 1 mm for the whole section and 20 µm for the selected zoom-ins. **(E)** Mice bearing luciferase-labeled Me275 tumors were harvested, and individual organs were analyzed by IVIS. Representative images of a control mouse and three hNSG-SGM3 mice at 8 wk after 10^6^ Me275 s.c. implantation. Tumor, s.c. implantation site. aLN, auxiliary LN; mesLN, mesenteric LN; nd, not done. **(F)** hNSG and hNSG-SGM3 mice were implanted with 10^6^ luciferase-labeled Me275 cells; tumor growth was monitored weekly by IVIS (*n* = 2 mice per group). **(G)** Mice bearing luciferase-labeled Me275 tumor were harvested at different time points, and individual organ was analyzed by IVIS. **(H)** Number of macroscopic tumors in the spleen and liver of NSG-SGM3 and hNSG-SGM3 mice implanted with 10^7^ Me275 cells s.c. *n* = 12–13 mice per strain, two-tailed Mann-Whitney test. **(I)** Localization of hCD45 (red), MART-1/gp100 (green), and DAPI (blue) in the liver from one mouse per strain from H. Scale bar = 1 mm for the whole section and 60 µm for the selected zoom-ins. **(J)** Outline of the experiment. 10^7^ hCD45^+^ cells enriched from naive hNSG-SGM3 mice were transferred i.v. into NSG-SGM3 or NSG mice and subsequently implanted with 10^7^ Me275 cells s.c. on the same day. **(K)** Macroscopic tumors in the liver at 8 wk after ACT as in J. *n* = 5 mice per recipient group, one-way ANOVA test. ****, P < 0.0001; ***, P < 0.001; **, P < 0.01. p, photons; sr, steradiance.

**Figure S2.**
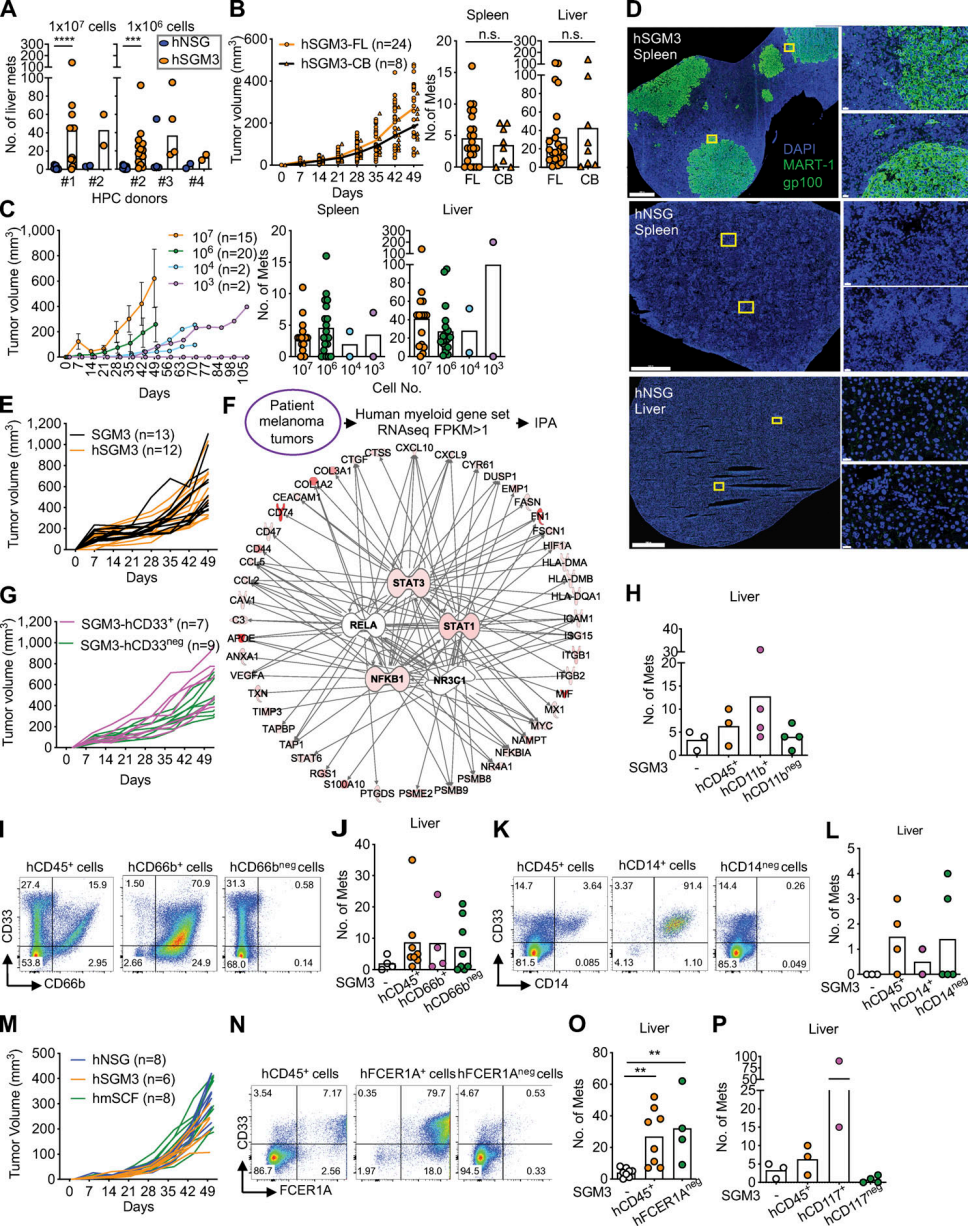
**hNSG-SGM3 mice promote melanoma growth in distant organs via hCD33^+^ cells.** **(A)** Macroscopic tumor in the liver of hNSG or hNSG-SGM3 reconstituted with different human fetal liver CD34^+^ HPC donors and implanted s.c. with 10^7^ or 10^6^ Me275 cells for 7–8 wk. *n* = 2–13 mice with two-tailed Mann-Whitney test. **(B)** Tumor growth and macroscopic tumor in the spleen and liver of hNSG-SGM3 reconstituted with either human fetal liver (FL) or cord blood (CB) HPCs and implanted s.c. with 10^6^ Me275 cells for 7–8 wk. *n* = 8–24 mice with two-tailed Mann-Whitney test. **(C)** Tumor growth and macroscopic tumor in the spleen and liver of hNSG-SGM3 mice reconstituted with human fetal liver HPCs and implanted s.c. with different number of Me275 cells. *n* = 8–24 mice. The error bar is the SD. **(D)** Localization of MART-1/gp100 (green) and DAPI (blue) in the spleen or liver of hNSG or hNSG-SGM3 at 8 wk after 10^7^ Me275 s.c. implantation. Scale bar = 1,000 µm for the whole section and 20 µm for the selected zoom-ins. **(E)** Primary tumor growth curves comparing NSG-SGM3 and hNSG-SGM3 mice implanted with 10^7^ Me275 cells s.c. *n* = 12–13 mice. **(F)** IPA analysis on myeloid genes from RNA-seq data in 14 metastatic patient melanoma tumors for the upstream regulator. Top five regulators, *STAT3*, *RELA*, *STAT1*, *NFKB1*, and *NR3C1*, are illustrated with the top 20 genes of each network, with the color intensity representing the magnitude of the expression. **(G)** Primary tumor growth curve in NSG-SGM3 mice after i.v. ACT of 10^7^ hCD33^+^ or hCD33^neg^ cells and subsequently implanted with 10^7^ Me275 cells s.c. *n* = 7–9 mice. **(H)** Macroscopic Me275 tumors in the livers of NSG-SGM3 mice after i.v. ACT of 10^7^ hCD11b^+^ or hCD11b^neg^ cells and subsequent s.c. implantation of 10^7^ Me275 cells. *n* = 3 or 4 mice per recipient group. **(I)** FACS plots illustrated the expression of hCD33 and CD66b on cells enriched for CD66b from naive hNSG-SGM3 mice for ACT experiment. **(J)** Macroscopic Me275 melanoma tumors in the livers of NSG-SGM3 mice after i.v. ACT of 10^7^ hCD45^+^, hCD66b^+^, or hCD66b^neg^ cells. *n* = 4–8 mice with one-way ANOVA test. **(K)** FACS plots illustrated the expression of hCD33 and CD14 on cells enriched for CD14 from naive hNSG-SGM3 mice for ACT experiment. **(L)** Macroscopic Me275 melanoma tumors in the livers of NSG-SGM3 mice after i.v. ACT of 10^7^ hCD45^+^, hCD14^+^, or hCD14^neg^ cells. *n* = 2–5 mice with one-way ANOVA test. **(M)** hNSG, hNSG-SGM3, and hNSG-mSCF (labeled hmSCF) mice were implanted with 10^6^ Me275 cells s.c. Primary tumor growth curves from two independent experiments using two CD34^+^ HPC donors. *n* = 6–8 mice. **(N)** FACS plots illustrated the expression of hCD33 and FCER1A on cells enriched for FCER1A from naive hNSG-SGM3 mice for ACT experiment. **(O)** Macroscopic Me275 melanoma tumor in the livers of NSG-SGM3 mice after i.v. ACT of 10^7^ hCD45^+^ or hFCER1A^neg^ cells. *n* = 4–8 mice with one-way ANOVA test. **(P)** Macroscopic Me275 tumors in the livers of NSG-SGM3 mice after ACT of 10^7^ hCD117^+^ or hCD117^neg^ cells i.v. and subsequent s.c. implantation of 10^7^ Me275 cells. *n* = 2–4 mice per recipient group. Mets, metastases. ****, P < 0.0001; ***, P < 0.001; **, P < 0.01.

We then implanted the two humanized mouse strains with 10^7^ Me275 human melanoma cells s.c. (Fig. 2 A; Rongvaux et al., 2014). Upon necropsy before any tumor-induced mortality, hNSG-SGM3 mice displayed a vastly greater number of macroscopic tumors in the spleen and liver than hNSG mice (Fig. 2 B; and Fig. S2, A–C), while implantation site s.c. tumors (primary tumors) grew at similar rates in both strains (Fig. 2 C). Immunofluorescence staining for melanoma-associated proteins, melanoma antigen recognized by T cells 1 (MART-1), and glycoprotein 100 (gp100) confirmed the presence of melanoma cells and tumor formation in distant visceral organs (Fig. 2 D and Fig. S2 D). Tumors were imaged in vivo or ex vivo with an in vivo imaging system (IVIS) to detect luciferase-labeled Me275 cells (Fig. 2, E and F). Luciferase signal was present in multiple visceral organs (liver, spleen, pancreas, stomach, kidney, lungs, and bone; Fig. 2 E). Kinetic experiments following the appearance of macroscopic tumors as well as luminescence signal reflecting tumor growth in vivo revealed that Me275 cells require at least 42 d for visceral tumors to appear (Fig. 2, F and G).

To determine if human leukocytes were involved, we compared Me275 tumor growth in NSG-SGM3 mice with or without a human immune system. Only sporadic macroscopic tumors in visceral organs could be found upon necropsy (Fig. 2 H), and no MART-1/gp-100 staining could be detected in the liver harvested from nonhumanized mice (Fig. 2 I). In contrast, distant organs were colonized with Me275 cells in hNSG-SGM3 mice (Fig. 2, H and I). In both cases, Me275 cells grew at the primary site at similar kinetics (Fig. S2 E). Thus, human leukocytes are critical for distant organ colonization by melanoma cells. This was further confirmed by adoptive cell transfer (ACT) experiments where hCD45^+^ leukocytes were purified from the liver and spleen of tumor-naive hNSG-SGM3 mice and adoptively transferred (10^7^ cells) into nonirradiated tumor-naive nonhumanized NSG-SGM3 mice or NSG mice. Both cohorts were implanted with Me275 cells s.c. immediately thereafter (Fig. 2 J). NSG-SGM3 mice adoptively transferred with hCD45^+^ leukocytes showed significantly higher numbers of melanoma tumors in the liver than controls without ACT (Fig. 2 K). In contrast, NSG mice adoptively transferred with hCD45^+^ cells purified from hNSG-SGM3 mice developed only sporadic liver tumors (Fig. 2 K). Thus, the capacity of hCD45^+^ leukocytes to support melanoma growth in distant organs is dependent on at least one of the host human cytokines SCF, GM-CSF, and/or IL-3.

### Distant organ colonization is dependent upon hCD33^+^ myeloid cells

**Figure 3.**
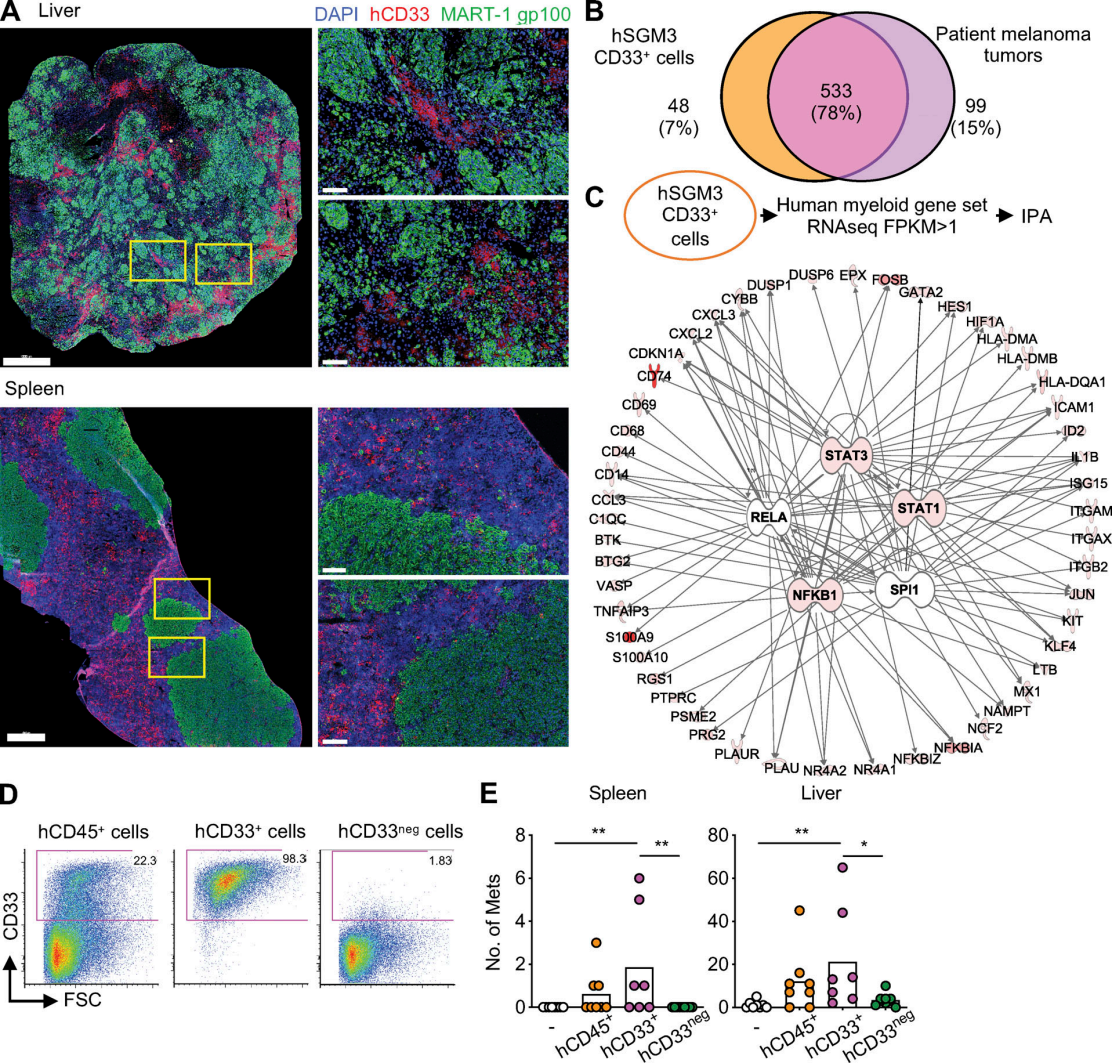
**Melanoma colonization in hNSG-SGM3 mice is dependent on hCD33^+^ cells. (A)** Immunofluorescence staining of MART-1/gp100 (green), CD33 (red), and DAPI (blue) in the liver and spleen of hNSG-SGM3 mice at 8 wk after 10^7^ Me275 s.c. implantation. Scale bar = 1 mm for the whole section and 100 µm for the selected zoom-ins. **(B)** Venn diagram illustrating human myeloid genes expressed in patient melanoma tumors and CD33^+^ cells in hNSG-SGM3 mice. **(C)** IPA on myeloid genes in CD33^+^ cells from hNSG-SGM3 mice for the upstream regulator. Top five regulators, *STAT3*, *RELA*, *STAT1*, *NFKB1*, and *SPI1*, are illustrated with the top 20 genes of each network. **(D)** Expression of hCD33 in enriched cells by FACS. **(E)** Macroscopic tumors in the spleen and liver of NSG-SGM3 mice after i.v. ACT of 10^7^ hCD33^+^ or hCD33^neg^ cells and subsequently implanted s.c. with 10^7^ Me275 cells. *n* = 7–9 mice per recipient group, one-way ANOVA test. FSC, forward scatter; Mets, metastases. **, P < 0.01.

Tissue examination revealed close proximity of hCD33^+^ cells and melanoma cells in the liver and spleen of hNSG-SGM3 mice (Fig. 3 A). The transcriptional profiles of hCD33^+^ cells purified from the spleen and liver of hNSG-SGM3 mice were established using RNA-seq, and the analysis was focused on the myeloid gene set selected by Nanostring. The Venn diagram analysis revealed a 78% overlap in myeloid gene expression between melanoma metastases from patients and hCD33^+^ cells from mice (Fig. 3 B). The top networks were driven by the expression of *STAT3*, *STAT1*, and *NFKB1,* identical to those observed in melanoma patients (Fig. 3 C, Fig. S2 F, Table S6, and Table S7). To establish whether hCD33^+^ myeloid cells are involved in tumor formation, hCD45^+^ cells isolated from the liver and spleen of hNSG-SGM3 mice were subdivided using magnetic beads into hCD33^+^ and hCD33^neg^ cell fractions (Fig. 3 D) and used in ACT experiments as described above. Recipient mice that received hCD33^+^ cells showed melanoma tumors in the spleen and in the liver, while those that received hCD33^neg^ cells did not (Fig. 3 E; with no impact on the growth of the primary tumors as shown in Fig. S2 G).

**Figure 4.**
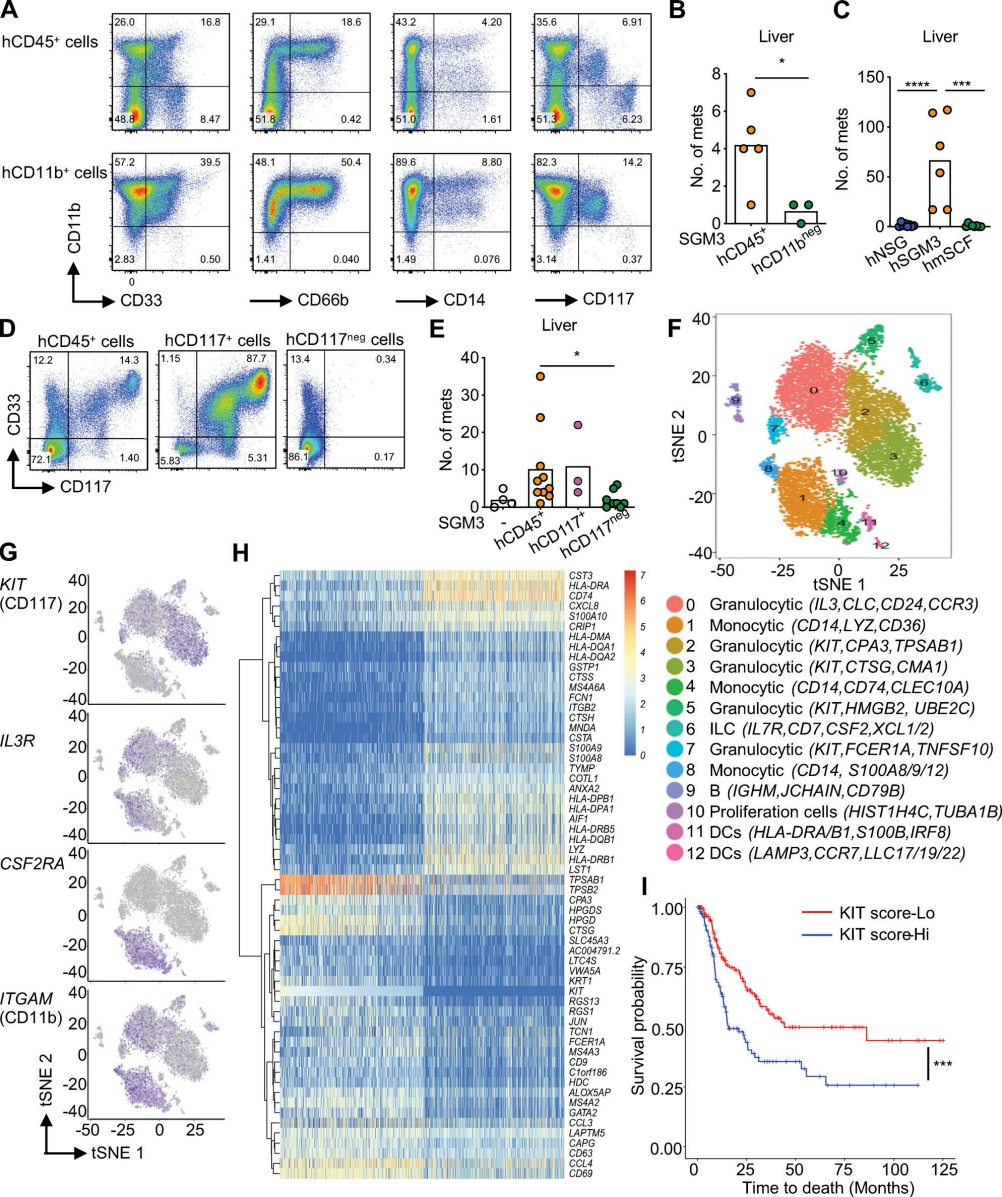
**hCD33^+^CD11b^+^CD117^+^ myeloid cells promote visceral organ colonization. (A)** Expression of hCD11b versus CD33, CD66b, CD14, and CD117 on enriched hCD45^+^ or CD11b^+^ cells from naive hNSG-SGM3 mice for ACT. **(B)** Macroscopic Me275 tumors in the livers of NSG-SGM3 mice after i.v. ACT of 10^7^ hCD45^+^ or hCD11b^neg^ cells and subsequent s.c. implantation of 10^7^ Me275 cells. *n* = 3–5 mice per recipient group, *t* test. **(C)** hNSG, hNSG-SGM3, and hNSG-mSCF (labeled hmSCF) mice were implanted s.c. with 10^6^ Me275 cells. Number of macroscopic tumors in the livers of mice from two independent experiments using two HPC donors. *n* = 6–8 mice per strain, one-way ANOVA test. **(D)** Expression of hCD33 and CD117 on cells enriched for CD117^+^ from naive hNSG-SGM3 mice for ACT. **(E)** Macroscopic Me275 tumors in the livers of NSG-SGM3 mice after ACT of 10^7^ hCD117^+^ or hCD117^neg^ cells i.v. and subsequent s.c. implantation of 10^7^ Me275 cells. *n* = 3–8 mice per recipient group, one-way ANOVA test. **(F)** scRNA-seq analysis of hCD33^+^ cells enriched from the spleen and liver of hNSG-SGM3 mice. 13 clusters with distinct transcriptional profiles are visualized using tSNE plot. ILC, innate lymphoid cells; DCs, dendritic cells. **(G)** *KIT-*, *IL3R-*, *CSF2RA-*, and *ITGAM*-expressing cells were color-mapped using tSNE plots. **(H)** DEGs on *KIT*^+^ cells (log expression ≥1) and *KIT*^neg^ cells (log expression = 0) from scRNA-seq of hCD33^+^ cells were analyzed using a mixture-modeling approach and rank ordered based on the log fold change. The top 30 up-regulated and down-regulated DEGs in *KIT^+^* cells are illustrated using the hierarchically clustered heatmap. **(I)** Kaplan-Meier analysis comparing survival of melanoma patients from TCGA stratified by median KIT score. P**= 0.00045 with the log-rank test. Mets, metastases. ****, P < 0.0001; ***, P < 0.001; *, P < 0.05.

hCD11b^+^ cells represent a large fraction of hCD33^+^ cells (71% ± 14%; range, 57–87%; *n* = 4; Fig. 4 A), and the ACT of CD11b-depleted CD45^+^ cells resulted in a lower number of liver tumors in a fraction of mice (0.7 ± 0.6 in the hCD11b^neg^ group; Fig. 4 B) while hCD11b^+^ cells could enhance liver tumors (12.8 ± 12.4, *n* = 4 mice; Fig. S2 H), suggesting that these cells play a role. hCD11b^+^ cells include CD66b^+^ cells that contain mature granulocytes and are present in hNSG-SGM3 but not in hNSG mice (Fig. S1 F), CD14^+^ cells of monocytic lineage, and cells with an intermediate expression of CD117 (Fig. 4 A). We did not observe any significant difference in liver tumors of mice transferred with CD66b-depleted cells or CD66b-enriched cells (Fig. S2, I and J), suggesting that CD66b^+^ cells are not essential for colonization of liver in hNSG-SGM3 mice. Similarly, depletion of CD14^+^ cells did not impact distant tumor development (Fig. S2, K and L), indicating that cells of monocytic lineage are dispensable. Thus, hCD33^+^ cells contain cell subset(s), distinct from mature granulocytes and monocytes, able to support melanoma growth in the liver.

### Distant organ colonization is mediated by hCD33^+^CD11b^+^CD117^+^ cells

hCD117^+^ (KIT*^+^*) cells were enriched in hNSG-SGM3 mice compared with hNSG (Fig. S1 F). The expression of CD117 within hCD33^+^ cells was heterogeneous, with at least three cell populations detectable by surface marker expression: CD33^+^CD11b^+^CD117^neg^, CD33^+^CD11b^+^CD117^+^, and CD33^+^CD11b^neg^CD117^+^ (Fig. 4 A). Because NSG mice expressing membrane-bound human SCF (NSG-mSCF) develop CD117^+^ tryptase^+^ mature human mast cells (Takagi et al., 2012), we constructed hNSG-mSCF mice and implanted them s.c. with 10^6^ Me275 cells. We did not detect tumors in the liver, and only sporadic spleen tumors in two of eight mice (Fig. 4 C), while there was a comparable primary tumor growth (Fig. S2 M). Furthermore, NSG-SGM3 mice with ACT of FCER1A-depleted hCD45^+^ cells displayed disseminated tumors in the liver (Fig. S2, N and O). Thus, hFCER1A^+^ (a marker of mature mast cells and basophils) cells are dispensable. However, depletion of hCD117^+^ cells resulted in a significant decrease in the number of mice with liver tumors as well as in the number of liver tumors per mouse in two independent experiments (1–35 in hCD45^+^ transferred mice, *n* = 11/13 vs. 0–6 in CD117-depleted hCD45^+^ transferred mice, *n* = 2/12; Fig. 4, D and E; and Fig. S2 P). Conversely, ACT of hCD117^+^ cells enriched from liver/spleen into nonhumanized NSG-SGM3 mice supported the development of liver tumors (range, 4–90) in the liver in five of five mice (Fig. 4 E and Fig. S2 P). Thus, the capacity to promote melanoma growth in distant organs lies within hCD33^+^CD11b^+^CD117^+^ precursor/progenitor cells, which represent ∼4% of the hCD45^+^ cells in hNSG-SGM3 mice.

**Figure S3.**
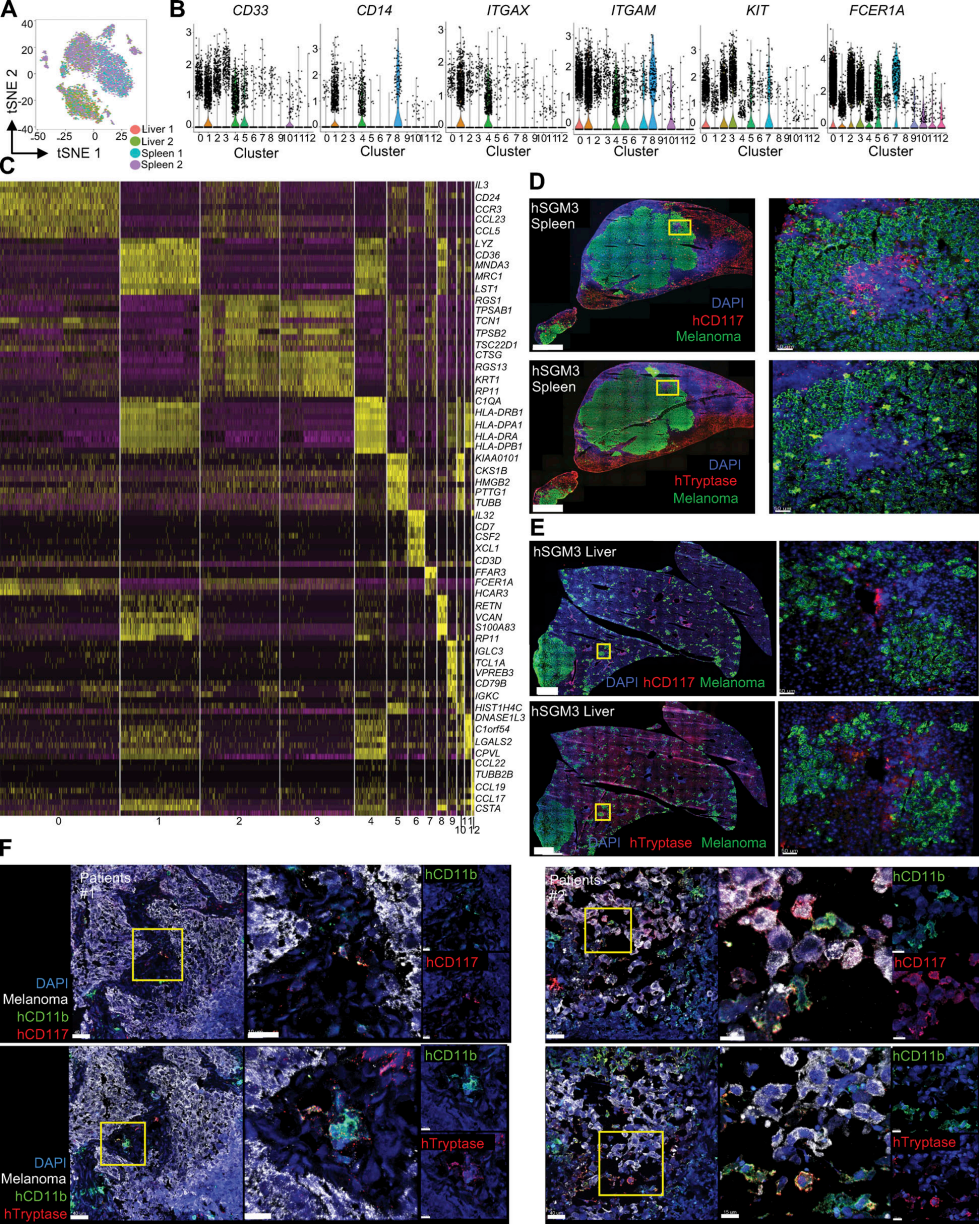
**The expression of CD117 in hNSG-SGM3 mice and metastatic melanoma tumors. (A)** hCD33^+^ cells from the spleen and liver of hNSG-SGM3 mice were subjected to scRNA-seq and tSNE reduction. Tissue localization from two independent experiments were color mapped into the tSNE map. **(B)** Violin plots showing the scRNA-seq expression value [log_2_ (FPKM +1)] of CD33 marker genes. **(C)** Expression of marker gene order by the clusters. **(D)** Immunofluorescence staining of human MART-1/gp100 melanoma cells (green), CD117 (upper panels, red) or Tryptase (lower panels, red), and DAPI (blue) in the spleen of hNSG-SGM3 mice at 8 wk after 10^7^ Me275 s.c. implantation. Scale bar = 1 mm for the whole section (left panels) and 100 µm for the selected zoom-ins (right panels). **(E)** Immunofluorescence staining in the liver of hNSG-SGM3 mice as in D.** (F)** Immunofluorescence staining of human MART-1/gp100 melanoma cells (white), CD11b (green), CD117 (upper panels, red), or Tryptase (lower panels, red), and DAPI (blue) in human melanoma tumors. Scale bar = 40 µm for the whole section (left panels) and 15 µm for the selected zoom-ins (right panels).

Single-cell RNA-seq (scRNA-seq) of hCD33^+^ cells from the liver/spleen of hNSG-SGM3 mice (Fig. 4 F; Fig. S3, A–C; and Table S8) revealed that *KIT*^+^ cells, which coexpress *ITGAM* (CD11b) and *IL3RA* but not *CSF2RA* (Fig. 4 G), are composed of four clusters (Fig. 4 F, clusters 2, 3, 5, and 7; and Fig. S3, B and C), including two clusters of cells expressing transcripts coding for mast cell proteolytic enzymes (*TPSAB1*,* TPSB2*) and leukotriene catabolism (*HPGD*, *HPGDS*, *LTC4S*; clusters 2 and 3); one cluster of mature mast cells with high expression of high-affinity IgE receptor (*FCER1A*) and *CCR3* (cluster 7); and one cluster of dividing progenitor cells expressing cell cycle and DNA synthesis genes (cluster 5). In line with this, tissue analysis revealed differences between CD117 and tryptase expression in both experimental metastatic tumors in hNSG-SGM3 mice and in metastasis from melanoma patients (Fig. S3, D–F). As the depletion of FCER1A-expressing cells did not impact melanoma growth in distant organs (Fig. S3 I), we conclude that pro-metastatic activity is distant from mature mast cells and is linked with progenitor cells.

We then stratified the cells into two groups based on the expression level of the *KIT* gene (Fig. 4 H) to establish the transcriptome of *KIT*^+^ cells. The differentially expressed genes (DEGs) between the two groups of cells were computed using the Wilcoxon rank sum test. All the genes that had an absolute log_2_ fold-change value >0.2 and a false discovery rate of <5% were selected as *KIT* signature genes (*n* = 221; Table S9). The prognostic significance was evaluated using the log_2_ fold-change weighted mean expression of the *KIT* signature genes in samples in TCGA (Cancer Genome Atlas Network, 2015). Since the score is a measure of the specific cell type, the samples were then stratified into two groups based on the median of the score, and the survival difference in the two groups was visualized using Kaplan-Meier plots (Fig. 4 I). The Kaplan-Meier analysis revealed that the high *KIT* score was associated with significantly lower survival probability (P**< 0.0005; hazard ratio = 1.94; 95% confidence interval = 1.33, 2.82).

Thus, hCD33^+^CD11b^+^CD117^+^ myeloid cells facilitate distant organ colonization by human melanoma after subcutaneous implantation. The molecular mechanisms regulating the ability of these cells to promote tumor growth in our model remain to be identified. Importantly, despite the presence of endogenous murine myeloid cells, the distant organ colonization is sporadic in the absence of human myeloid cells. This suggests that the molecular pathways governing myeloid cell–dependent melanoma growth are restricted in the absence of human cells. Our studies herein suggest a potential novel prognostic biomarker and downstream effector molecule(s) that might represent a therapeutic target.

## Materials and methods

### Cell lines

Melanoma cancer cell line Me275 (Research Resource Identifier [RRID]:CVCL_S597), which was established from surgically excised melanoma metastases from patient LAU50, was provided by Pedro Romero at the Ludwig Institute for Cancer Research at the University of Lausanne (Lausanne, Switzerland). Tumor cells were cultured in complete RPMI (RPMI 1640, 25 mM Hepes, 1 mM sodium pyruvate, 1% nonessential amino acid, 1% penicillin-streptomycin, and 2 mM L-glutamine) supplemented with 10% FBS at 37°C with 5% CO_2_ atmosphere and authenticated using Short Tandem Repeat profiling analysis by the American Type Culture Collection. The mycoplasma test was performed regularly, and cells were negative for mycoplasma before each experiment.

### Lentiviral transduction

The Me275 tumor cell lines were transduced with lentiviral vector (pLX302-CMV-Luc2) at varying multiplicity of infection by incubating virions in the culture medium containing 8 µg/ml polybrene (Sigma). Stably transduced cells were selected with 3 µg/ml puromycin (InvivoGen). Stable cell lines expressing luciferase protein were confirmed by anti-luciferase (Luci21 1–107; Novus) intracellular staining.

### Humanized mice

Humanized mice were generated on NSG (NOD.Cg-Prkdc^scid^ Il2rg^tm1Wjl^/SzJ; RRID:IMSR JAX:005557), NSG-SGM3 (NOD.Cg-Prkdc^scid^ Il2rg^tm1Wjl^Tg(CMV-IL3,CSF2,KITLG)1Eav/MloySzJ; RRID:IMSR JAX:013062), and NSG-mSCF (NOD.Cg-Prkdc^scid^ Il2rg^tm1Wjl^ Tg(PGK1-KITLG*220)441Daw/SzJ; RRID:IMSR JAX:017830) obtained from The Jackson Laboratory. All protocols were reviewed and approved by the Institutional Animal Care and Use Committee at The Jackson Laboratory (14005) and University of Connecticut Health Center (101163–0220). De-identified human specimens were obtained from vendors and approved by The Jackson Laboratory Institutional Review Board. Mice were sublethally irradiated (10 cGy per gram of body weight) using γ irradiation at the age of 4 wk. 100,000 CD34^+^ HPCs from fetal liver or full-term cord blood (Advanced Bioscience Resources or Lonza) were given by tail-vein i.v. injection in 200 µl PBS. Mice were bled at 8–12 wk after HPC transplant to evaluate engraftment and were euthanized according to the individual experimental design.

### Flow cytometry analysis

For human engraftment, heparinized blood samples were first treated with RBC lysis buffer (Biolegend), followed by the treatment of both human and murine Fc Block (BD Biosciences) and then stained on ice with antibodies to mouse CD45 (30-F11; BD Biosciences) and hCD45 (HI30; BD Biosciences), CD14 (MqP9; BD Biosciences) or CD33 (P67.6; Biolegend), CD19 (HIB19; Biolegend), and CD3 (SK7; BD Biosciences) for 30 min (Table S10). Antibodies to mouse and hCD45 were species-specific antibodies to pan-leukocytes including myeloid cells. After washing twice with PBS, the samples were acquired on an LSRII or Symphony A5 (BD Biosciences) and analyzed with FlowJo software (Tree Star). For immunophenotype, bone marrow (femur and tibia), spleen, livers, lungs, and blood were collected. Tissues were first digested with 25 µg/ml Liberase (Roche Diagnostics) and DNase I (Sigma) at 37°C, 10 min for the spleen and 30 min for livers and lungs. Single-cell suspensions were made, and the debris was removed by filtering through a 70-µm cell strainer. Cells were first treated with RBC lysis buffer, followed by the treatment of both human and murine Fc Block, and then stained on ice with fluorescence-conjugated antibodies to mouse CD45 (30-F11; BD Biosciences) and hCD45 (HI30; BD Biosciences), CD3 (OKT3; BD Biosciences), CD19 (HIB19; Biolegend) or CD20 (2H7; Biolegend), CD33 (P67.6; Biolegend), CD11b (ICRF44; Biolegend), CD11c (B-ly6; BD Biosciences), CD14 (MqP9; BD Biosciences), CD16 (3G8; BD Biosciences), CD66b (G10F5; Biolegend), CD117 (104D2; Biolegend), CD127 (HIL-7R-M21; BD Biosciences), FCER1A (AER-37; Biolegend), and HLA-DR (G46-6; BD Biosciences) for 30 min (Table S10). After washing twice with PBS, the samples were stained with 7-aminoactinomycin D (Biolegend) and acquired on LSRII or Symphony A5 (BD Biosciences) and analyzed with FlowJo software (Tree Star).

### Tumor model

Tumor cells were injected s.c. into the flank of the mice. Tumor size was monitored every 7 d with a caliper. Tumor volume (ellipsoid) was calculated as follows: (short diameter)^2^ × long diameter/2. Alternatively, luciferase-labeled melanoma cells were injected i.v. into the mice. Mice were killed, and the macroscopic metastases were identified and scored in various organs including lymph nodes (axillary and brachial), livers, spleen, kidneys, and lungs.

### Human immune cell purification and ACT

At 12–16 wk after transplant, hNSG-SGM3 mice were euthanized, and spleen and liver were collected for single-cell suspension. Spleen and liver were first digested with 25 µg/ml Liberase (Roche Diagnostics) for 10–30 min at 37°C; single-cell suspensions were made, and the debris was removed by filtering through a 70-µm cell strainer. Live cells were isolated using Ficoll-Paque Plus density gradient centrifugation. Human immune cells were enriched by using the Mouse/Human Chimera isolation kit (StemCell Technologies) following the manufacturer’s protocol. For the enrichment of CD33^+^ or CD33^neg^ cells, enriched hCD45^+^ cells were further stained with PE-conjugated CD33 antibody (WM53; Biolegend) for 15 min and enriched by using EasySep PE selection kit (StemCell Technologies). For CD14, CD11b, CD66b, CD117, and FCER1A depletion, hCD45^+^ cells were further stained with PE-conjugated CD14 (MϕP-9; BD Biosciences), CD11b (IRCF44; Biolegend), CD66b (G10F5; Biolegend), CD117 (104D2; Biolegend), and FCER1A (AER-37; Biolegend) antibodies (Table S10) for 15 min and enriched by using the EasySep PE selection kit (StemCell Technologies). Isolated human immune cells had purity exceeding 95%. Human immune cells were adoptively transferred at 10^7^ cells per mouse by tail-vein i.v. injection.

### In vivo imaging

Before mice were anesthetized with Isoflurane, an aqueous solution of luciferin (150 mg/kg i.p.) was injected 10 min before imaging with IVIS (PerkinElmer). The animals were placed into the light-tight chamber of the charge-coupled device camera system, and the photons emitted from the luciferase-expressing cells within the animal were quantified using Living Image (PerkinElmer). To image dissected organs, mice were first injected i.p. with luciferin (150 mg/kg) for 10 min and quickly killed to remove each organ. Organs were imaged in 12-well culture dishes with PBS containing 300 µg/ml luciferin.

### Immunofluorescence staining

Tissues were embedded in optimal cutting temperature media (Sakura Finetek U.S.A.) and snap-frozen in liquid nitrogen. Frozen sections were cut at 6 µm, air-dried on Superfrost plus slides, and fixed with cold acetone for 5 min. Tissue sections were first blocked with Background Buster, followed by treatment of Fc Receptor Block (Innovex Bioscience). The sections were then stained with mouse monoclonal antibodies to human MART-1 (M2-2C10 and M2-9E3; Novus Biologicals), gp100 (NK1-beteb; LifeSpan BioSciences), CD45 (HI30; Biolegend), CD33 (P67.6; Biolegend), CD11b (ICRF44), CD117 (104D2; Biolegend), and tryptase (AA1; Biolegend) for 1 h at room temperature (Table S10). Respective isotype antibodies were used as the control. Finally, sections were counterstained with 1 µg/ml of DAPI, mounted with Fluoromount (Thermo Fisher Scientific), acquired using a Leica SP 8 confocal microscope with Leica LAS X software, and analyzed using Imaris software (Bitplane).

### RNA-seq

Total RNA was isolated from snap-frozen metastatic melanoma tissues (Table S1; Cooperative Human Tissue Network, Pennsylvania) and CD33^+^ cells from the spleen and livers of hNSG-SGM3 and hNSG mice using the RNA isolation kit following the manufacturer’s protocol (Qiagen). Total RNA isolated was run on a Qubit (Thermo Fisher Scientific) and a Bioanalyzer 2100 Nano Chip (Agilent Technologies) to check RNA quantity and quality. Sequencing libraries were prepared using KAPA Stranded mRNA-seq kit (Roche) according to the manufacturer’s protocol. First, poly-A RNA was isolated from 300 ng total RNA using oligo-dT magnetic beads. Purified RNA was then fragmented at 85°C for 6 min, targeting fragment range 250–300 bp. Fragmented RNA was reverse transcribed with an incubation of 25°C for 10 min and 42°C for 15 min and an inactivation step at 70°C for 15 min. This was followed by second strand synthesis at 16°C for 60 min. Double-stranded cDNA fragments were purified using Ampure XP beads (Beckman Coulter), then A-tailed and ligated with Illumina adapters. Adapter-ligated DNA was purified using AMPure XP beads and followed by 10 cycles of PCR amplification. The final library was cleaned up using AMPure XP beads. Quantification of libraries was performed using real-time quantitative PCR (Thermo Fisher Scientific). Sequencing was performed on an Illumina NextSeq 500 platform generating single-end reads of 75 bp. All primary analysis of RNA-seq was processed using CASAVA pipeline (Illumina, v1.8.2). Sequences were aligned with Bowtie 2 (Kim et al., 2013), and counts were generated with RSEM (Anders et al., 2015) using the annotations from Ensembl GRCh37 (Harrow et al., 2012). The files from alignment result were converted to BAM format using SAMtools (Li et al., 2009). Raw counts were normalized to log_2_ transformed transcript per million (TPM) or fragments per kilobase of transcript per million mapped reads (FPKM; log_2_(FPKM + 1)). CIBERSORT was used to estimate the proportions of diverse immune cell types using the genes that define the signature expression of the immune cell types. We used the default 22 cell types (LM22) provided (Newman et al., 2015). For modular analysis, a set of 260 transcriptional modules was used as a preexisting framework (Banchereau et al., 2016). Module-level activity score was calculated by R package gene set variation analysis (GSVA) score from RNA-seq data (Hänzelmann et al., 2013). Ingenuity pathway analysis (IPA; Qiagen) was applied to reveal transcriptional networks, and ClueGO was used to illustrate biological interpretation of genes (Bindea et al., 2009).

### scRNA-seq

Enriched hCD33^+^ cells from liver and spleen of hNSG-SGM3 mice were resuspended in PBS containing 0.04% BSA, the cell numbers were counted on the Contess II automated cell counter (Thermo Fisher Scientific), and ∼12,000 cells were loaded per channel on Chromium microfluidic chips (10x Genomics). Single-cell capture, barcoding, and library preparation were performed using the 10x Chromium version 2 chemistry according to the manufacturer’s protocol (10x Genomics). The quality of cDNA and libraries was checked on an Agilent 4200 TapeStation, quantified by KAPA quantitative PCR, and sequenced on a HiSeq 4000 (Illumina) to an average depth of 50,000 reads per cell. We quantified gene expression counts from raw sequencing data using Cell Ranger v2.2 with GRCh38. Datasets from liver and spleen of two independent experiments were normalized using Harmony (Korsunsky et al., 2019).

### Database and statistical analysis

Statistical analysis was performed in Prism 8 (GraphPad). Figure legends denote P values as follows: ****, P < 0.0001; ***, P < 0.001; **, P < 0.01; and *, P < 0.05. Comparisons between any two groups were analyzed using the Mann-Whitney test or two-tailed *t* test, and comparisons between any three or more groups were analyzed by ANOVA as indicated in the respective legends. The Kaplan-Meier curves of melanoma patient data were generated using RNA-seq data from the TCGA–skin cutaneous melanoma project (Cancer Genome Atlas Network, 2015). The DEGs between the two groups of cells were computed using the Wilcoxon rank sum test. The statistical significance of the difference in the Kaplan-Meier survival plot was computed using the log-rank test using R package.

### Data availability

RNA-seq data have been deposited at the NCBI Sequence Read Archive with accession no. SRP141256 (https://www.ncbi.nlm.nih.gov/Traces/study1/?acc=SRP141256&go=go), and scRNA-seq data have been deposited in the NCBI Gene Expression Omnibus with accession no. GSE161957 and Sequence Read Archive with accession no. SRP293689 (https://www.ncbi.nlm.nih.gov/Traces/study1/?acc=SRP293689&go=go).

### Online supplemental material

Fig. S1 shows human myeloid cells in metastatic melanoma tumors and hNSG-SGM3 mice. Fig. S2 shows that hNSG-SGM3 mice promote melanoma growth in distant organs via hCD33^+^ cells. Fig. S3 shows the expression of hCD117 in hNSG-SGM3 mice and metastatic melanoma tumor. Table S1 shows the list of melanoma patient tumors used in the study. Table S2 shows the list of genes in annotated immune modules. Table S3 shows the kinetics of human engraftment in the blood of hNSG and hNSG-SGM3 mice. Table S4 shows human engraftment in the tissues of hNSG and hNSG-SGM3 mice. Table S5 shows DEGs of bulk RNA-seq on hCD33^+^ cells enriched from the spleen and liver of hNSG and hNSG-SGM3 mice. Table S6 shows IPA on upstream regulator for the myeloid genes expressed in CD33^+^ cells from hNSG-SGM3 mice. Table S7 shows IPA on upstream regulator for the myeloid genes expressed in human melanoma tumors. Table S8 shows marker genes specific for each cluster in scRNA-seq of hCD33^+^ cells from hNSG-SGM3 mice. Table S9 shows *KIT* signature genes identified from scRNA-seq of hCD33^+^ cells from hNSG-SGM3 mice. Table S10 shows the list of antibodies used in the study.

## Supplementary Material

Table S1 lists the melanoma patient tumors used in the study.

Table S2 lists the genes in the annotated immune modules.

Table S3 shows the kinetics of human engraftment in the blood of hNSG and hNSG-SGM3 mice.

Table S4 shows human engraftment in the tissues of hNSG and hNSG-SGM3 mice.

Table S5 shows DEGs of bulk RNA-seq on hCD33^+^ cells enriched from the spleen and liver of hNSG and hNSG-SGM3 mice.

Table S6 shows the IPA on upstream regulators for the myeloid genes expressed in CD33^+^ cells from hNSG-SGM3 mice.

Table S7 shows the IPA on upstream regulators for the myeloid genes expressed in human melanoma tumors.

Table S8 shows marker genes specific for each cluster in scRNA-seq of hCD33^+^ cells from hNSG-SGM3 mice.

Table S9 shows *KIT* signature genes identified from scRNA-seq of hCD33^+^ cells from hNSG-SGM3 mice.

Table S10 lists the antibodies used in the study.

## References

[bib1] Acharyya, S., T. Oskarsson, S. Vanharanta, S. Malladi, J. Kim, P.G. Morris, K. Manova-Todorova, M. Leversha, N. Hogg, V.E. Seshan, . 2012. A CXCL1 paracrine network links cancer chemoresistance and metastasis. Cell. 150:165–178. 10.1016/j.cell.2012.04.042 22770218 PMC3528019

[bib2] Anders, S., P.T. Pyl, and W. Huber. 2015. HTSeq--a Python framework to work with high-throughput sequencing data. Bioinformatics. 31:166–169. 10.1093/bioinformatics/btu638 25260700 PMC4287950

[bib3] Balch, C.M., J.E. Gershenwald, S.J. Soong, J.F. Thompson, M.B. Atkins, D.R. Byrd, A.C. Buzaid, A.J. Cochran, D.G. Coit, S. Ding, . 2009. Final version of 2009 AJCC melanoma staging and classification. J. Clin. Oncol. 27:6199–6206. 10.1200/JCO.2009.23.4799 19917835 PMC2793035

[bib4] Banchereau, R., S. Hong, B. Cantarel, N. Baldwin, J. Baisch, M. Edens, A.M. Cepika, P. Acs, J. Turner, E. Anguiano, . 2016. Personalized Immunomonitoring Uncovers Molecular Networks that Stratify Lupus Patients. Cell. 165:551–565. 10.1016/j.cell.2016.03.008 27040498 PMC5426482

[bib5] Bekes, E.M., B. Schweighofer, T.A. Kupriyanova, E. Zajac, V.C. Ardi, J.P. Quigley, and E.I. Deryugina. 2011. Tumor-recruited neutrophils and neutrophil TIMP-free MMP-9 regulate coordinately the levels of tumor angiogenesis and efficiency of malignant cell intravasation. Am. J. Pathol. 179:1455–1470. 10.1016/j.ajpath.2011.05.031 21741942 PMC3157227

[bib6] Billerbeck, E., W.T. Barry, K. Mu, M. Dorner, C.M. Rice, and A. Ploss. 2011. Development of human CD4+FoxP3+ regulatory T cells in human stem cell factor-, granulocyte-macrophage colony-stimulating factor-, and interleukin-3-expressing NOD-SCID IL2Rγ(null) humanized mice. Blood. 117:3076–3086. 10.1182/blood-2010-08-301507 21252091 PMC3062310

[bib7] Bindea, G., B. Mlecnik, H. Hackl, P. Charoentong, M. Tosolini, A. Kirilovsky, W.H. Fridman, F. Pagès, Z. Trajanoski, and J. Galon. 2009. ClueGO: a Cytoscape plug-in to decipher functionally grouped gene ontology and pathway annotation networks. Bioinformatics. 25:1091–1093. 10.1093/bioinformatics/btp101 19237447 PMC2666812

[bib8] Cancer Genome Atlas Network. 2015. Genomic Classification of Cutaneous Melanoma. Cell. 161:1681–1696. 10.1016/j.cell.2015.05.044 26091043 PMC4580370

[bib9] Coffelt, S.B., K. Kersten, C.W. Doornebal, J. Weiden, K. Vrijland, C.S. Hau, N.J.M. Verstegen, M. Ciampricotti, L.J.A.C. Hawinkels, J. Jonkers, and K.E. de Visser. 2015. IL-17-producing γδ T cells and neutrophils conspire to promote breast cancer metastasis. Nature. 522:345–348. 10.1038/nature14282 25822788 PMC4475637

[bib10] Datta, M., L.M. Coussens, H. Nishikawa, F.S. Hodi, and R.K. Jain. 2019. Reprogramming the Tumor Microenvironment to Improve Immunotherapy: Emerging Strategies and Combination Therapies. Am. Soc. Clin. Oncol. Educ. Book. 39:165–174. 10.1200/EDBK_237987 31099649 PMC6596289

[bib11] Eggermont, A.M., V. Chiarion-Sileni, J.J. Grob, R. Dummer, J.D. Wolchok, H. Schmidt, O. Hamid, C. Robert, P.A. Ascierto, J.M. Richards, . 2016. Prolonged Survival in Stage III Melanoma with Ipilimumab Adjuvant Therapy. N. Engl. J. Med. 375:1845–1855. 10.1056/NEJMoa1611299 27717298 PMC5648545

[bib12] Einhorn, L.H., M.A. Burgess, C. Vallejos, G.P. Bodey Sr., J. Gutterman, G. Mavligit, E.M. Hersh, J.K. Luce, E. Frei III, E.J. Freireich, and J.A. Gottlieb. 1974. Prognostic correlations and response to treatment in advanced metastatic malignant melanoma. Cancer Res. 34:1995–2004. 4842252

[bib13] Etzerodt, A., K. Tsalkitzi, M. Maniecki, W. Damsky, M. Delfini, E. Baudoin, M. Moulin, M. Bosenberg, J.H. Graversen, N. Auphan-Anezin, . 2019. Specific targeting of CD163^+^ TAMs mobilizes inflammatory monocytes and promotes T cell-mediated tumor regression. J. Exp. Med. 216:2394–2411. 10.1084/jem.20182124 31375534 PMC6781002

[bib42] Flaherty, Keith, Michael A. Davies, Jean Jacques Grob, Georgina V. Long, Paul D. Nathan, Antoni Ribas, Caroline Robert, Dirk Schadendorf, Dennie T Frederick, Marc R Hammond, . 2016. Genomic analysis and 3-y efficacy and safety update of COMBI-d: A phase 3 study of dabrafenib (D) + trametinib (T) vs D monotherapy in patients (pts) with unresectable or metastatic BRAF V600E/K-mutant cutaneous melanoma. JCO. 34(15_suppl):9502–9502. 10.1200/JCO.2016.34.15_suppl.9502

[bib14] Garner, H., and K.E. de Visser. 2020. Immune crosstalk in cancer progression and metastatic spread: a complex conversation. Nat. Rev. Immunol. 20:483–497. 10.1038/s41577-019-0271-z 32024984

[bib15] Gupta, T.D., and R. Brasfield. 1964. Metastatic Melanoma. A Clinicopathological Study. Cancer. 17:1323–1339. 10.1002/1097-0142(196410)17:10<1323::AID-CNCR2820171015>3.0.CO;2-N 14236766

[bib16] Hagai, T., X. Chen, R.J. Miragaia, R. Rostom, T. Gomes, N. Kunowska, J. Henriksson, J.E. Park, V. Proserpio, G. Donati, . 2018. Gene expression variability across cells and species shapes innate immunity. Nature. 563:197–202. 10.1038/s41586-018-0657-2 30356220 PMC6347972

[bib17] Hänzelmann, S., R. Castelo, and J. Guinney. 2013. GSVA: gene set variation analysis for microarray and RNA-seq data. BMC Bioinformatics. 14:7. 10.1186/1471-2105-14-7 23323831 PMC3618321

[bib18] Harrow, J., A. Frankish, J.M. Gonzalez, E. Tapanari, M. Diekhans, F. Kokocinski, B.L. Aken, D. Barrell, A. Zadissa, S. Searle, . 2012. GENCODE: the reference human genome annotation for The ENCODE Project. Genome Res. 22:1760–1774. 10.1101/gr.135350.111 22955987 PMC3431492

[bib19] Hodi, F.S., S.J. O’Day, D.F. McDermott, R.W. Weber, J.A. Sosman, J.B. Haanen, R. Gonzalez, C. Robert, D. Schadendorf, J.C. Hassel, . 2010. Improved survival with ipilimumab in patients with metastatic melanoma. N. Engl. J. Med. 363:711–723. 10.1056/NEJMoa1003466 20525992 PMC3549297

[bib20] Jaillon, S., A. Ponzetta, D. Di Mitri, A. Santoni, R. Bonecchi, and A. Mantovani. 2020. Neutrophil diversity and plasticity in tumour progression and therapy. Nat. Rev. Cancer. 20:485–503. 10.1038/s41568-020-0281-y 32694624

[bib21] Kanazawa, N. 2007. Dendritic cell immunoreceptors: C-type lectin receptors for pattern-recognition and signaling on antigen-presenting cells. J. Dermatol. Sci. 45:77–86. 10.1016/j.jdermsci.2006.09.001 17046204

[bib22] Kim, D., G. Pertea, C. Trapnell, H. Pimentel, R. Kelley, and S.L. Salzberg. 2013. TopHat2: accurate alignment of transcriptomes in the presence of insertions, deletions and gene fusions. Genome Biol. 14:R36. 10.1186/gb-2013-14-4-r36 23618408 PMC4053844

[bib23] Korsunsky, I., N. Millard, J. Fan, K. Slowikowski, F. Zhang, K. Wei, Y. Baglaenko, M. Brenner, P.R. Loh, and S. Raychaudhuri. 2019. Fast, sensitive and accurate integration of single-cell data with Harmony. Nat. Methods. 16:1289–1296. 10.1038/s41592-019-0619-0 31740819 PMC6884693

[bib24] Lewis, C.E., A.S. Harney, and J.W. Pollard. 2016. The Multifaceted Role of Perivascular Macrophages in Tumors. Cancer Cell. 30:18–25. 10.1016/j.ccell.2016.05.017 27411586 PMC5024543

[bib25] Li, H., B. Handsaker, A. Wysoker, T. Fennell, J. Ruan, N. Homer, G. Marth, G. Abecasis, and R. Durbin. 1000 Genome Project Data Processing Subgroup. 2009. The Sequence Alignment/Map format and SAMtools. Bioinformatics. 25:2078–2079. 10.1093/bioinformatics/btp352 19505943 PMC2723002

[bib41] Long, G.V., K.T. Flaherty, D. Stroyakovskiy, H. Gogas, E. Levchenko, F. de Braud, J. Larkin, C. Garbe, T. Jouary, A. Hauschild, . 2017. Dabrafenib plus trametinib versus dabrafenib monotherapy in patients with metastatic BRAF V600E/K-mutant melanoma: long-term survival and safety analysis of a phase 3 study. Ann Oncol. 28(7):1631–1639. 10.1093/annonc/mdx176 28475671 PMC5834102

[bib26] Mantovani, A., F. Marchesi, A. Malesci, L. Laghi, and P. Allavena. 2017. Tumour-associated macrophages as treatment targets in oncology. Nat. Rev. Clin. Oncol. 14:399–416. 10.1038/nrclinonc.2016.217 28117416 PMC5480600

[bib27] Mestas, J., and C.C. Hughes. 2004. Of mice and not men: differences between mouse and human immunology. J. Immunol. 172:2731–2738. 10.4049/jimmunol.172.5.2731 14978070

[bib28] Meyer, J.E., and L. Stolbach. 1978. Pretreatment radiographic evaluation of patients with malignant melanoma. Cancer. 42:125–126. 10.1002/1097-0142(197807)42:1<125::AID-CNCR2820420121>3.0.CO;2-7 667789

[bib29] Nathanson, L., T.C. Hall, and S. Farber. 1967. Biological aspects of human malignant melanoma. Cancer. 20:650–655. 10.1002/1097-0142(1967)20:5<650::AID-CNCR2820200512>3.0.CO;2-5 6024277

[bib30] Newman, A.M., C.L. Liu, M.R. Green, A.J. Gentles, W. Feng, Y. Xu, C.D. Hoang, M. Diehn, and A.A. Alizadeh. 2015. Robust enumeration of cell subsets from tissue expression profiles. Nat. Methods. 12:453–457. 10.1038/nmeth.3337 25822800 PMC4739640

[bib31] Nozawa, H., C. Chiu, and D. Hanahan. 2006. Infiltrating neutrophils mediate the initial angiogenic switch in a mouse model of multistage carcinogenesis. Proc. Natl. Acad. Sci. USA. 103:12493–12498. 10.1073/pnas.0601807103 16891410 PMC1531646

[bib32] Ouzounova, M., E. Lee, R. Piranlioglu, A. El Andaloussi, R. Kolhe, M.F. Demirci, D. Marasco, I. Asm, A. Chadli, K.A. Hassan, . 2017. Monocytic and granulocytic myeloid derived suppressor cells differentially regulate spatiotemporal tumour plasticity during metastatic cascade. Nat. Commun. 8:14979. 10.1038/ncomms14979 28382931 PMC5384228

[bib33] Patel, J.K., M.S. Didolkar, J.W. Pickren, and R.H. Moore. 1978. Metastatic pattern of malignant melanoma. A study of 216 autopsy cases. Am. J. Surg. 135:807–810. 10.1016/0002-9610(78)90171-X 665907

[bib34] Rongvaux, A., T. Willinger, J. Martinek, T. Strowig, S.V. Gearty, L.L. Teichmann, Y. Saito, F. Marches, S. Halene, A.K. Palucka, . 2014. Development and function of human innate immune cells in a humanized mouse model. Nat. Biotechnol. 32:364–372. 10.1038/nbt.2858 24633240 PMC4017589

[bib35] Ruffell, B., and L.M. Coussens. 2015. Macrophages and therapeutic resistance in cancer. Cancer Cell. 27:462–472. 10.1016/j.ccell.2015.02.015 25858805 PMC4400235

[bib36] Spiegel, A., M.W. Brooks, S. Houshyar, F. Reinhardt, M. Ardolino, E. Fessler, M.B. Chen, J.A. Krall, J. DeCock, I.K. Zervantonakis, . 2016. Neutrophils Suppress Intraluminal NK Cell-Mediated Tumor Cell Clearance and Enhance Extravasation of Disseminated Carcinoma Cells. Cancer Discov. 6:630–649. 10.1158/2159-8290.CD-15-1157 27072748 PMC4918202

[bib37] Szczerba, B.M., F. Castro-Giner, M. Vetter, I. Krol, S. Gkountela, J. Landin, M.C. Scheidmann, C. Donato, R. Scherrer, J. Singer, . 2019. Neutrophils escort circulating tumour cells to enable cell cycle progression. Nature. 566:553–557. 10.1038/s41586-019-0915-y 30728496

[bib38] Takagi, S., Y. Saito, A. Hijikata, S. Tanaka, T. Watanabe, T. Hasegawa, S. Mochizuki, J. Kunisawa, H. Kiyono, H. Koseki, . 2012. Membrane-bound human SCF/KL promotes in vivo human hematopoietic engraftment and myeloid differentiation. Blood. 119:2768–2777. 10.1182/blood-2011-05-353201 22279057 PMC3327455

[bib39] Williams, A., R.A. Flavell, and S.C. Eisenbarth. 2010. The role of NOD-like Receptors in shaping adaptive immunity. Curr. Opin. Immunol. 22:34–40. 10.1016/j.coi.2010.01.004 20149616 PMC7038629

[bib40] Wolchok, J.D., V. Chiarion-Sileni, R. Gonzalez, P. Rutkowski, J.J. Grob, C.L. Cowey, C.D. Lao, J. Wagstaff, D. Schadendorf, P.F. Ferrucci, . 2017. Overall Survival with Combined Nivolumab and Ipilimumab in Advanced Melanoma. N. Engl. J. Med. 377:1345–1356. 10.1056/NEJMoa1709684 28889792 PMC5706778

