# Supplementary material for: Human KIT+ myeloid cells facilitate visceral metastasis by melanoma
Source: J Exp Med. 2021 Apr 15;218(6):e20182163. doi: 10.1084/jem.20182163 (PMC8056753; doi:10.1084/jem.20182163)
Supplement: Table S1 — lists the melanoma patient tumors used in the study. [file JEM_20182163_TableS1.docx]

Table S1. List of melanoma patient tumors used in the study.

| Serial No | Tumor ID | Age | Gender | Race | Stage | Site | Treatments |
| --- | --- | --- | --- | --- | --- | --- | --- |
| T1 | 52067T001 | 87 | F | W | MET | Metastatic axillary node | No |
| T2 | 59818T003 | 60 | M | U* | MET | Metastatic jejunum | Radiation |
| T3 | 63362T001 | 79 | M | W | MET | Lung | No |
| T4 | 63804T005 | 65 | M | W | MET | Metastatic axillary node | No |
| T5 | 67135T002 | 57 | M | W | MET | Metastatic axillary node | Radiation |
| T6a  T6b | 68352T005; 68352T006^#^ | 50 | M | W | MET | Metastatic axillary node | No |
| T7 | 75319T003 | 30 | M | W | MET | Skin, back | Dabrafenib, Trametinib |
| T8 | 75664T003 | 63 | F | W | MET | Soft tissue | PD1 |
| T9 | 75687T001 | 60 | M | W | MET | Metastatic ileo-inguinal node | No |
| T10 | 75955T003 | 65 | M | W | MET | Metastatic axillary node | Ipilimumab |
| T11 | 76283T003 | 77 | F | U | MET | Metastatic iliac node | No |
| T12 | 76484T004 | 61 | M | W | MET | Adrenal gland | Unknown |
| T13 | 77956T001 | 71 | M | W | MET | Metastatic axillary node | Unknown |
| T14 | 77958T001 | 81 | M | W | MET | Metastatic axillary node | Unknown |

* Unknown; ^#^ Two different region collected from the same patient tumor.
