# Supplementary material for: Human KIT+ myeloid cells facilitate visceral metastasis by melanoma
Source: J Exp Med. 2021 Apr 15;218(6):e20182163. doi: 10.1084/jem.20182163 (PMC8056753; doi:10.1084/jem.20182163)
Supplement: Table S3 — shows the kinetics of human engraftment in the blood of hNSG and hNSG-SGM3 mice. [file JEM_20182163_TableS3.docx]

Table S3. The kinetics of human engraftment in the blood of hNSG and hNSG-SGM3 mice.

| Time | Strains | hCD45 (%) | hCD14 (%) | hCD19 (%) | hCD3 (%) |
| --- | --- | --- | --- | --- | --- |
| 4-wk | hNSG | 4.2±1.5 | 32.4±8.8 | 18.4±3.6 | 0 |
|  | hNSG-SGM3 | 52.6±6.4*** | 43.8±1.5* | 4.8±0.8 | 0.05±0.03 |
| 6-wk | hNSG | 24.9±4.3 | 3.3±0.7 | 78.5±2.3 | 0.07±0.03 |
|  | hNSG-SGM3 | 72.4±5.7*** | 31.6±3.2**** | 38.7±4.2*** | 0.13±0.03 |
| 8-wk | hNSG | 31.6±10.2 | 2.0±0.3 | 86.8±2.5 | 0.8±1.2 |
|  | hNSG-SGM3 | 79.1±5.5*** | 23.7±4.8*** | 48.3±5.9*** | 1.8±2.2 |
| 10-wk | hNSG | 36.2±9.0 | 2.2±0.3 | 87.4±4.1 | 3.5±4.0 |
|  | hNSG-SGM3 | 86.7±7.8*** | 21.1±2.0*** | 23.3±13.2**** | 29.2±18.0** |
| 12-wk | hNSG | 43.6±4.1 | 6.5±1.2 | 59.2±8.4 | 13.2±10.0 |
|  | hNSG-SGM3 | 92.6±6.8*** | 11.4±0.8 | 17.7±9.5*** | 28.5±10.6 |

hCD45 (%) represents the percentage of hCD45^+^ cells in total h+mCD45^+^ cells. hCD14 (%), hCD19 (%) and hCD3 (%) represent the percentage in hCD45^+^ cells. Data represent mean±SD from 3 mice with 2way ANOVA and Bonferroni’s multiple comparison test between hNSG and hNSG-SGM3.
