# Supplementary material for: Human KIT+ myeloid cells facilitate visceral metastasis by melanoma
Source: J Exp Med. 2021 Apr 15;218(6):e20182163. doi: 10.1084/jem.20182163 (PMC8056753; doi:10.1084/jem.20182163)
Supplement: Table S4 — shows human engraftment in the tissues of hNSG and hNSG-SGM3 mice. [file JEM_20182163_TableS4.docx]

Table S4. Human engraftment in the tissues of hNSG and hNSG-SGM3 mice.

| Tissues | Strains | hCD45  (% & cell no.) | hCD33  (% & cell no.) | hCD19  (% & cell no.) | hCD3  (% & cell no.) |
| --- | --- | --- | --- | --- | --- |
| Bone marrow | hNSG | 93.3±2.5%  3.08±0.23E+7 | 42.8±6.2%  1.27±0.22E+4 | 45±5.7%  1.33±0.17E+4 | 0.14±0.1%  4.35±3.2E+4 |
|  | hNSG-SGM3 | 97.3±2.1%  2.08±0.2E+7* | 70.4±0.8%****  1.45±0.15E+4 | 16.2±3.3%****  3.34±0.8E+3* | 11.3±6.0%*  2.3±1.15E+6 |
| Spleen | hNSG | 93.7±1.5%  1.46±0.57E+7 | 4.2±0.5%  6.07±2.67E+2 | 79.1±2.4%  1.13±0.46E+4 | 5.0±3.0%  8.15±7.25E+5 |
|  | hNSG-SGM3 | 96.3±1.5%  1.41±0.04E+8**** | 12.5±2.4%*  1.77±0.38E+4**** | 40.4±5.1%****  5.7±0.53E+4**** | 33.1±4.0%****  4.67±0.66E+7**** |

hCD45 (%) represents the percentage of hCD45^+^ cells in total h+mCD45^+^ cells. hCD14 (%), hCD19 (%) and hCD3 (%) represent the percentage in hCD45^+^ cells. Data represent mean±SD from 3 mice with 2way ANOVA and Bonferroni’s multiple comparison test between hNSG and hNSG-SGM3.
