# Supplementary material for: Human KIT+ myeloid cells facilitate visceral metastasis by melanoma
Source: J Exp Med. 2021 Apr 15;218(6):e20182163. doi: 10.1084/jem.20182163 (PMC8056753; doi:10.1084/jem.20182163)
Supplement: Table S6 — shows the IPA on upstream regulators for the myeloid genes expressed in CD33+ cells from hNSG-SGM3 mice. [file JEM_20182163_TableS6.docx]

Table S6. IPA on upstream regulator for the myeloid genes expressed in CD33^+^ cells from hNSG-SGM3 mice.

| Upstream regulator | p-value of overlap | Target molecules in dataset |
| --- | --- | --- |
| *STAT3* | 1.22E-72 | *ALOX15,AREG,ARG1,BATF,BCL2,BCL6,BIRC5,C5AR1,CASP1,CASP7,CCL2,CCL20,CCL4,CCL5,CCR1,CCR5,CCRL2,CD209,CD274,CD40,CD74,CD80,CD86,CDH1,CDKN1A,CEACAM1,CEBPA,CEBPB,CEBPD,CSF2,CTSL,CXCL10,CXCL2,CXCL3,CXCL8,CXCL9,CXCR2,CXCR3,CXCR4,DPP4,EGR2,EGR3,FAS,FASN,FCGR1A,FLT1,FN1,FSCN1,FUT4,GATA3,GPR65,HGF,HIF1A,HIST2H2AA3/HIST2H2AA4,HLA-DMA,HLA-DQA1,ICAM1,ID2,IFIT1,IKBKE,IL10,IL12B,IL13,IL18R1,IL1B,IL1R1,IL1RN,IL23A,IL4R,IL6,IL6R,IRF1,IRF4,IRF5,IRF7,ISG15,ITGAM,ITGB1,ITGB2,JAG1,KLF4,LIF,LTA,MAFB,MMP9,MX1,MX2,MYC,MYD88,NAMPT,NFATC2,NFKB1,NFKBIZ,NR4A2,PIM2,PLAU,PLAUR,PROK2,PSMB8,PSMB9,PTAFR,PTGS2,S100A9,SERPINB9,SOCS1,SOCS3,STAT1,STAT3,TAP1,TGFB1,THBD,TLR3,TNF,TNFRSF1B,TNFSF10,USP18,VEGFA* |
| *RELA* | 7.95E-68 | *ALCAM,ALOX5AP,APOE,BCL10,BCL2,BIRC2,BIRC3,BIRC5,BTG2,C3,CAMP,CCL19,CCL2,CCL20,CCL22,CCL3,CCL5,CCR7,CD14,CD40,CD44,CD69,CD80,CDKN1A,CEBPB,CSF2,CXCL1,CXCL10,CXCL11,CXCL2,CXCL3,CXCL5,CXCL8,CXCL9,CXCR4,CYBB,DUSP1,FAS,FN1,FOSB,FSCN1,GCH1,HES1,ICAM1,IER3,IKBKE,IL10,IL12B,IL13,IL15RA,IL1A,IL1B,IL1RN,IL23A,IL4,IL6,IRF1,IRF4,IRF7,ISG15,JUN,KIT,KLF10,LTA,LTB,MIF,MMP9,MYC,NAMPT,NFATC1,NFKB1,NFKBIA,NFKBIE,NOD2,NR4A1,NR4A2,OLR1,PLAU,PPARG,PSMB9,PTGDS,PTGS2,PTX3,SMAD7,STAT5A,TAP1,TAP2,TAPBP,TERF2IP,TGFB1,TGM2,TLR2,TNF,TNFAIP3,TNFRSF4,TRAF1,TREM1,VASP,VCAM1,VEGFA* |
| *STAT1* | 5.48E-64 | *ALOX15,APOE,ARG1,BIRC5,C3,C4A/C4B,CASP1,CCL19,CCL2,CCL20,CCL3,CCL5,CCR6,CCR7,CCRL2,CD14,CD274,CD40,CD86,CDKN1A,CEACAM1,CEBPD,CREM,CSF2,CSF3R,CTSS,CX3CR1,CXCL10,CXCL11,CXCL2,CXCL3,CXCL8,CXCL9,CXCR3,DPP4,FAS,FCGR1A,FCGR2B,FURIN,GATA3,HAVCR2,HES1,HIF1A,HLA-DQA1,ICAM1,IDO1,IFIT1,IL10,IL12B,IL15,IL15RA,IL1B,IL1R1,IL4,IL6,IRF1,IRF2,IRF5,IRF7,IRF8,ISG15,ITGAX,JUN,KLF4,MMP9,MX1,MYC,PDCD1LG2,PDGFA,PPARG,PSMB8,PSMB9,PSME2,PTGS2,S100A10,SMAD2,SMAD7,SOCS1,SOCS3,STAT1,STAT3,TAP1,TLR3,TLR4,TLR8,TNF,TNFSF10,TRAF2,TRAFD1,USP18* |
| *NFKB1* | 1.55E-56 | *APOE,BCL2,BIRC3,BTG2,CCL19,CCL2,CCL20,CCL22,CCL4,CCL5,CD40,CD80,CD86,CDKN1A,CSF1,CSF2,CXCL10,CXCL11,CXCL2,CXCL3,CXCL8,CXCL9,CXCR5,CYBB,DUSP1,ENPP2,FAS,FOSB,FSCN1,GATA3,GNAI3,HLA-DMB,ICAM1,ICOSLG/LOC102723996,IER3,IKBKE,IL10,IL12B,IL13,IL18,IL1B,IL1RN,IL23A,IL6,IRF1,IRF4,ISG15,JAG1,LTA,LTB,MMP9,MYC,NFATC1,NFKB1,NFKBIA,NOD2,NR4A1,NR4A2,PDGFA,PLAU,PTAFR,PTGS2,PTX3,RGS1,STAT1,TGFB1,TLR2,TNF,TNFAIP3,TNFRSF4,TNFSF10,TRAF1,TRAF2,VCAM1,VEGFA* |
| *SPI1* | 2.69E-54 | *ADGRE1,BCL6,BTK,C1QC,C3AR1,CCL17,CCL22,CCL3,CCL4,CCR6,CCR7,CD14,CD180,CD209,CD68,CDKN1A,CEBPA,CSF1,CSF1R,CSF2RA,CSF3R,CTSS,CXCR1,CYBB,DUSP6,EGR2,ELANE,EPX,FCER1A,FCGR2B,GATA1,GATA2,HES1,ID2,ID3,IKZF1,IL10,IL12B,IL13,IL18,IL1B,IL1R2,IL1RN,IL4,IRF4,ISG15,ITGA4,ITGA5,ITGAM,ITGB2,JUN,KIT,KLF4,MS4A1,MYC,NCF2,OSCAR,PIK3CG,PRG2,PSMB8,PSMB9,PTGS2,PTPRC,TLR2,TLR4,TNF,TNFRSF14,TREM1,VAV1* |
