# Supplementary material for: Human KIT+ myeloid cells facilitate visceral metastasis by melanoma
Source: J Exp Med. 2021 Apr 15;218(6):e20182163. doi: 10.1084/jem.20182163 (PMC8056753; doi:10.1084/jem.20182163)
Supplement: Table S7 — shows the IPA on upstream regulators for the myeloid genes expressed in human melanoma tumors. [file JEM_20182163_TableS7.docx]

Table S7. IPA on upstream regulator for the myeloid genes expressed in human melanoma tumors.

| Upstream regulator | p-value of overlap | Target molecules in dataset |
| --- | --- | --- |
| *STAT3* | 7.06E-77 | *ANGPT2,AREG,BATF,BCL2,BCL6,BIRC5,C5AR1,CASP1,CASP7,CCL2,CCL20,CCL4,CCL5,CCR1,CCR5,CCRL2,CD209,CD274,CD40,CD40LG,CD74,CD80,CD86,CDH1,CDH2,CDH5,CDKN1A,CEACAM1,CEBPA,CEBPB,CEBPD,COL1A2,COL3A1,CTGF,CTLA4,CTSL,CX3CL1,CXCL10,CXCL13,CXCL2,CXCL3,CXCL8,CXCL9,CXCR3,CXCR4,DPP4,EDN1,EGR2,EGR3,FAS,FASN,FCGR1A,FLT1,FN1,FSCN1,FUT4,GATA3,GPR65,HGF,HIF1A,HIST2H2AA3/HIST2H2AA4,HLA-DMA,HLA-DQA1,ICAM1,ICOS,ID2,IFIT1,IFNG,IKBKE,IL10,IL1B,IL1R1,IL1RN,IL4R,IL6,IL6R,IRF1,IRF4,IRF5,IRF7,ISG15,ITGAM,ITGB1,ITGB2,JAG1,KLF4,KLRC4-KLRK1/KLRK1,LIF,LTA,MAFB,MMP13,MMP9,MX1,MX2,MYC,MYD88,NAMPT,NFATC2,NFKB1,NFKBIZ,NR4A2,PIM2,PLAU,PLAUR,PSMB8,PSMB9,PTAFR,PTGS2,S100A9,SAA1,SERPINB9,SERPINE1,SOCS1,SOCS3,STAT1,STAT3,TAP1,TGFB1,THBD,TLR3,TNF,TNFRSF1B,TNFSF10,USP18,VEGFA* |
| *RELA* | 8.76E-70 | *ALCAM,ALOX5AP,APOE,BCL10,BCL2,BIRC2,BIRC3,BIRC5,BTG2,C3,CAV1,CCL19,CCL2,CCL20,CCL22,CCL3,CCL5,CCR7,CD14,CD40,CD44,CD69,CD80,CDKN1A,CEBPB,COL1A2,CTGF,CX3CL1,CXCL1,CXCL10,CXCL11,CXCL2,CXCL3,CXCL8,CXCL9,CXCR4,CYBB,DUSP1,EDN1,FAS,FGF2,FN1,FOSB,FSCN1,GCH1,HES1,ICAM1,IER3,IFNG,IGF1,IKBKE,IL10,IL15RA,IL1B,IL1RN,IL6,IRF1,IRF4,IRF7,ISG15,JUN,KIT,KLF10,LTA,LTB,MIF,MMP1,MMP9,MYC,NAMPT,NFATC1,NFKB1,NFKBIA,NFKBIE,NOD2,NR4A1,NR4A2,OLR1,PDGFB,PLAU,PPARG,PSMB9,PTGDS,PTGS2,PTX3,SAA1,SELE,SELP,SMAD7,STAT5A,TAP1,TAP2,TAPBP,TERF2IP,TGFB1,TGM2,TLR2,TNF,TNFAIP3,TNFRSF4,TRAF1,TREM1,VASP,VCAM1,VEGFA* |
| *STAT1* | 5.37E-63 | *ANGPT2,APOE,BIRC5,C3,C4A/C4B,CASP1,CCL19,CCL2,CCL20,CCL3,CCL5,CCR6,CCR7,CCRL2,CD14,CD274,CD40,CD86,CDKN1A,CEACAM1,CEBPD,CREM,CSF3R,CTSS,CX3CL1,CXCL10,CXCL11,CXCL2,CXCL3,CXCL8,CXCL9,CXCR3,DPP4,EDN1,FAS,FCGR1A,FCGR2B,FGF2,FGF7,FURIN,GATA3,HAVCR2,HES1,HIF1A,HLA-DQA1,ICAM1,IDO1,IFIT1,IFNG,IGF1,IL10,IL15,IL15RA,IL1B,IL1R1,IL6,IRF1,IRF2,IRF5,IRF7,IRF8,ISG15,ITGAX,JUN,KLF4,MMP9,MX1,MYC,NOX4,PDCD1LG2,PDGFA,PPARG,PSMB8,PSMB9,PSME2,PTGS2,S100A10,SMAD2,SMAD7,SOCS1,SOCS3,STAT1,STAT3,TAP1,TLR3,TLR4,TLR8,TNF,TNFSF10,TRAF2,TRAFD1,USP18* |
| *NFKB1* | 1.22E-58 | *APOE,BCL2,BIRC3,BTG2,CCL19,CCL2,CCL20,CCL22,CCL4,CCL5,CD40,CD80,CD86,CDKN1A,COL1A2,CSF1,CTGF,CX3CL1,CXCL10,CXCL11,CXCL2,CXCL3,CXCL8,CXCL9,CXCR5,CYBB,CYR61,DUSP1,ENPP2,FAS,FOSB,FSCN1,GATA3,GNAI3,HLA-DMB,ICAM1,ICOSLG/LOC102723996,IER3,IFNG,IKBKE,IL10,IL18,IL1B,IL1RN,IL6,IRF1,IRF4,ISG15,JAG1,LTA,LTB,MMP13,MMP9,MYC,NFATC1,NFKB1,NFKBIA,NOD2,NR4A1,NR4A2,PDGFA,PLAU,PTAFR,PTGS2,PTX3,RGS1,SELE,SELP,STAT1,TGFB1,TLR2,TNF,TNFAIP3,TNFRSF4,TNFSF10,TRAF1,TRAF2,VCAM1,VEGFA* |
| *NR3C1* | 1.39E-58 | *ALOX5AP,ANXA1,APOE,BCL10,BCL2,BCL6,BIRC2,BIRC3,BTK,C4A/C4B,CASP10,CASP7,CAV1,CCL2,CCL4,CCL5,CD38,CD40LG,CD47,CD69,CD70,CD83,CDKN1A,CEBPA,CEBPB,CRABP2,CXCL10,CXCL2,CXCL3,CXCL8,CXCR4,CYR61,DAXX,DUSP1,EDN1,EMP1,ENC1,FADD,FN1,GADD45B,GATA3,GEM,GPR65,GZMA,ICAM1,IER3,IGF1,IL10,IL15,IL15RA,IL18,IL1B,IL1RAP,IL3RA,IL6,INSR,IRF1,IRF8,ISG15,ITGB2,JUN,LIF,LTB,MALT1,MAP3K14,MMP1,MMP13,MMP8,MMP9,MYC,MYD88,NAMPT,NFATC1,NFIL3,NFKB1,NFKBIA,NLRP3,NOD2,OLR1,PDCD1LG2,PDGFA,PHLDA2,PPARG,PRKCI,PTGS2,RAF1,RAPGEF4,RIPK2,RUNX2,SELE,SERPINB9,SERPINE1,SGPP1,SMAD1,STAT3,STAT5A,STAT6,TGFB1,THBD,TIMP3,TLR1,TLR2,TLR5,TNF,TNFAIP3,TNFAIP6,TNFAIP8,TNFRSF12A,TNFRSF1A,TNFRSF1B,TNFRSF8,TNFRSF9,TNFSF4,TNFSF9,TRAF1,TUBA4A,TXN,VCAM1* |
