# Supplementary material for: Human KIT+ myeloid cells facilitate visceral metastasis by melanoma
Source: J Exp Med. 2021 Apr 15;218(6):e20182163. doi: 10.1084/jem.20182163 (PMC8056753; doi:10.1084/jem.20182163)
Supplement: Table S10 — lists the antibodies used in the study. [file JEM_20182163_TableS10.docx]

**Table S10**. List of antibodies used in the study.

| Antigen | Clone or references | Source |
| --- | --- | --- |
| Human CD3 | SK7 | BD Bioscience |
| Human CD3 | OKT3 | BD Bioscience |
| Human CD11b | ICRF44 | Biolegend |
| Human CD11c | B-Ly6 | BD Bioscience |
| Human CD14 | MqP9 | BD Bioscience |
| Human CD16 | 3G8 | Biolegend |
| Human CD19 | HIB19 | Biolegend |
| Human CD20 | 2H7 | Biolegned |
| Human CD33 | WM53 | Biolegend |
| Human CD33 | P67.6 | Biolegend |
| Human CD45 | HI30 | BD Bioscience |
| Human CD66b | G10F5 | Biolegend |
| Human CD117 | 104D2 | Biolegend |
| Human CD127 | HIL-7R-M21 | BD Bioscience |
| Human FCER1A | AER-37 | Biolegend |
| Human gp100 | NK1-beteb | LifeSpan BioSciences |
| Human HLA-DR | G46-6 | BD Bioscience |
| Human MART-1 | M2-2C10 and M2-9E3 | Novus Biological |
| Human Tryptase | AA1 | Biolegend |
| Mouse CD45 | 30-F11 | BD Bioscience |
| Mouse IgG1 isotype | MG1-45 | Biolegend |
| Mouse IgG2b isotype | MPC-11 | Biolegend |
| Goat anti-mouse-IgG1-Alexa 555-F(ab’)_2_ | A-21127 | Thermo Fisher Scientific |
| Goat anti-mouse-IgG2b-Alexa 488-F(ab’)_2_ | A-21141 | Thermo Fisher Scientific |
| Goat anti-mouse-IgG2b-Alexa 647-F(ab’)_2_ | A-21242 | Thermo Fisher Scientific |
| Goat anti-rabbit-IgG-Alexa 568-F(ab’)_2_ | A-11011 | Thermo Fisher Scientific |
| Rabbit IgG isotype | X090302-8 | Agilent |
